# Acoustic approach as an alternative to human-based survey in bird biodiversity monitoring in agricultural meadows

**DOI:** 10.1371/journal.pone.0266557

**Published:** 2022-04-08

**Authors:** Michał Budka, Marek Jobda, Paweł Szałański, Hubert Piórkowski

**Affiliations:** 1 Faculty of Biology, Department of Behavioural Ecology, Adam Mickiewicz University in Poznań, Poznań, Poland; 2 Department of Nature Protection and Rural Landscape, Institute of Technology and Life Sciences, Falenty, Poland; US Department of Agriculture, UNITED STATES

## Abstract

Acoustic monitoring has been tested as an alternative to the traditional, human-based approach of surveying birds, however studies examining the effectiveness of different acoustic methods sometimes yield inconsistent results. In this study we examined whether bird biodiversity estimated by traditional surveys of birds differs to that obtained through soundscape surveys in meadow habitats that are of special agricultural importance, and whether acoustic monitoring can deliver reliable indicators of meadows and farmland bird biodiversity. We recorded soundscape and simultaneously surveyed birds by highly skilled human-observers within a fixed (50 m and 100 m) and unlimited radius using the point-count method twice in the breeding season at 74 recording sites located in meadows, in order to compare differences in (1) bird biodiversity estimation of meadow, farmland, songbird, and all bird species and (2) the detection rate of single bird species by these two methods. We found that recorders detected more species in comparison to the human-observers who surveyed birds within a fixed radius (50 and 100 m) and fewer when detection distance for human-observers was unlimited. We did not find significant differences in the number of meadow and farmland bird species detected by recorders and observers within a 100 m radius–the most often used fixed radius in traditional human based point-counts. We also showed how detection rate of 48 the most common bird species in our study differ between these two methods. Our study showed that an acoustic survey is equally effective as human observers surveying birds within a 100 m radius in estimation of farmland and meadow bird biodiversity. These groups of species are important for agricultural landscape and commonly used as indicators of habitat quality and its changes. Even though recorders rarely detect species that remain mostly silent during the observation periods, or species that are further distant than 100 m away, we recommend using acoustic soundscape recording methods as an equally effective and more easily standardised alternative for monitoring of farmland and meadow bird biodiversity. We propose adaptation of acoustic approach to long-term, large-scale monitoring by collecting acoustic data by non-specialists, including landowners and volunteers, and analysing them in a standardised way by units supervising monitoring of agriculture landscape.

## Introduction

Birds are useful biological indicators of environmental conditions and their changes [1,2]. Therefore, conservation biologists and ecologists monitor bird populations in various environments, from a regional to a global spatial scale [3,4]. In the traditional human-based approach, highly skilled observers survey birds in the field by detecting and recognising them aurally and visually [5]. The most restrictive monitoring schemes require multiple surveys of study area done by observers with many years of experience in order to detect all territories and minimise the observer’s effect on survey results [6]. However, the number of highly skilled observers in a short breeding season is limited, and so this type of monitoring is usually conducted on a small, local scale. The large-scale monitoring schemes such as regional or national often include less experienced observers who possess varied skill sets and usually survey the study area twice in the breeding season, which entails a compromise between data quality and the spatial extent of monitoring.

Acoustic monitoring has been proposed as an effective alternative for traditional, observer-based approach [7–12]. The acoustic monitoring process combines data collection in the field with laboratory analyses of soundscape recordings [10,13,14]. An advantage of acoustic monitoring is that data can be collected in a highly standardised way in many locations at the same time [10], without highly skilled fieldworkers, observer effect, mistakes made in terms of species detection and identification in the field [15,16]. The acoustic monitoring is also low-cost in comparison to traditional observed-based approaches and allows for the generation of more data [10,17]. The final advantage is that original data are collected and stored, enabling the verification of correctness of species detections or for the re-analysis of recordings in the future using new detection methods and techniques [10]. Due to the possibility of repeated listening or spectrogram scanning, there is less chance to overlook the species or make an identification error [18], which is possible in traditional, human-based field surveys, especially when many species vocalise at the same time and often are observed for a short time [19,20].

Acoustic monitoring also has a few limitations, which should be considered. The first is that some bird species vocalise rarely or are completely silent during some parts of a day or season, thus cannot be detected by a recorder, while a human-observer is able to detect them visually [19,21]. However, this limitation can be easily broken by increasing the duration of the recording time or appropriate sampling across a day and season [22,23]. The second is the diversity of equipment used for soundscape recording [10,24]. Using recorders and microphones of different quality leads to the same sound recorded in the same habitat and weather conditions being recorded from a different distance by various recorders, thus sampling area is inconstant [24]. Another issue is the variation in amplitude and frequency of sounds produced by birds. Birds, depending on the species, produce sounds in wide range of frequency, from several dozen Hz to even 10 kHz [25]. Low-frequency sounds are generally transmitted for longer distance than high-frequency ones, thus considerable between-species variation in detection range is observed [24]. Independently of the frequency of sound, the loudness of birds’ vocalisation ranges from ca 50 to even 120 dB measured at 1 m [26,27]. These two characteristics of different bird species vocalisations result in the detection range being species-specific [28]. Moreover, the detection range strongly depends on habitat type—probability of species detection at a given distance is higher in open vegetation than in forest [29]. Therefore, it is important to know how effective the acoustic technique is in a specific environment inhabited by a composition of bird species which are characterised by various probabilities of acoustic detection and various distances from which the recorder can detect their vocalisations.

Comparisons of species detection by observer and recorder have been conducted around the world, mostly in forest or semi-forest habitats [19,21,23,30,31]. In most cases, researchers compared bird species richness from the same point and at the same time by both acoustic recorder and an observer who surveyed birds using a point count method. However, these various studies have delivered inconsistent results: from a higher species richness recorded by an observer [18,32], no statistically significant differences in species richness estimated by both methods [33,34], to a higher species richness detected by a recorder [21]. These disagreements may arise from a few methodological limitations, like differently skilled observers involved in such comparisons, habitat type causing differences in detection probabilities by recorder and observer, or distance categories (or unlimited radius) from which birds are recorded by observers during field surveys [19,23,24,30,35–37]. Therefore, to effectively compare bird biodiversity estimation by human observers and acoustic recorders, studies conducted by highly skilled observers, in a relatively homogenous habitat are required. By applying various detection distance categories for human observers and comparing them with detections made by recorders we may estimate the detection distance of individual species or groups for recorders and find potential species composition for which recorders and observers work equally well. In this way we make possible comparison of results of our study both with studies applying limited and unlimited distance of detection by human observers, and we show how using fixed or unlimited detection distance by human observer changes interpretation of results comparing biodiversity estimation by recorders and observers surveying birds at the same time.

In our study we asked whether acoustic monitoring can be an effective alternative for traditional, human-based approach to bird biodiversity monitoring in relatively homogeneous open habitat–agricultural landscape of meadows. Permanent grasslands and meadows covered 34% of the utilised agricultural area in the EU in 2016 (59 million hectares) [38] and are a key habitat for European birds [39]. The species inhabiting them, together with other farmland birds, are among the group of birds whose populations are rapidly declining [40]. Many European Union countries implement agri-environmental schemes (AES) to protect meadow habitats and their biodiversity [41]. Thus, simple, repeatable, and effective, large-scale monitoring of farmland bird species is needed to understand how birds respond to changes in agricultural management and how the conservation activities being undertaken affect particular species and their groups [42–44].

Here we compared similarities and differences of acoustic and traditional surveys by skilled, experienced observers in estimating biodiversity in Central European meadows over distances ≤ 50 m, ≤ 100 m, and unlimited range. The field methodology followed methods currently used for evaluation of effectiveness of agricultural measures dedicated for bird protection in Poland. We analysed the usefulness of acoustic recorders for biodiversity estimations of various groups of birds: (1) all bird biodiversity, (2) songbird biodiversity, (3) meadow bird biodiversity and (4) farmland bird biodiversity. We also examined which of the most common bird species are underdetected and which are overdetected by recorders in comparison to human-observers, to show potential, species-specific limitations of acoustic method. The goal of our study was to examine whether data collected by highly skilled ornithologists and acoustic recorders in meadows give similar estimation of overall bird biodiversity and biodiversity of species important for agricultural landscape—an environment in which large-scale, long-term acoustic monitoring could be used as an alternative approach to human-based bird surveys. This could result in establishing a monitoring scheme which enables non-specialists, including landowners, to collect field data which that can be analysed in standardized ways to augment traditional survey approaches.

## Materials and methods

### Fieldwork

We selected 74 recording sites located in meadows in Poland (see S1 Table for exact locations of each recording site). In each recording site, we visually estimated coverage by main habitat types and visibility (i.e., percent of the area within 100 m radius which can be visually inspected by observer). Meadows presented the main type of habitat within a 100 m radius around the recording sites (86%, SD = 18.6), while forests and buffer strips covered 8% (SD = 11.6%), arable fields 3% (SD = 12.0%), water reservoirs 3% (SD = 7.4%) of radius area (mean coverage of each habitat type around recording site and standard deviation (SD) are given). An average visibility within 100 m radius around a recording site was 85% (SD = 14.7%) and ranged from 40% to 100%.

At each recording site highly skilled fieldworker (MJ or PS) surveyed birds using the point-count method [45]. During a 10-minute survey, all birds seen or heard were assigned to one of three distance categories: ≤ 50 m, ≤ 100 m, and ≥ 100 m. Observers were trained to estimate distance before they start field surveys. At each recording site we conducted two 10-minute surveys during the 2019 breeding season: early survey (from April 11 to May 20; hours from 05:08 to 09:53 AM, local time) and late survey (from May 27 to June 30; hours from 05:01 to 08:59 AM, local time). The observations were noted in a previously prepared paper field forms. Such field methodology is commonly applied to evaluate effectiveness of agricultural measures dedicated to birds’ protection in Poland. Additionally, during each survey the soundscape (10 minutes of recording done while the human observer surveyed birds) was recorded using a portable digital recorder (Zoom H1n; two different recorders were used during the study) with a built-in microphone (unidirectional condenser, 90° XY stereo format) with windscreen. The quality of the recorder used is comparable with other models of autonomous sound recorders commonly used for soundscape recording [24]. The recorder was put horizontally on a tripod at 2 m above ground level and kept at an average of 5 m from the fieldworker. We recorded WAV audio files with sampling rate 48 kHz/16 bit. The same settings of recording were used throughout the study (input level: -10 dB; low-cut filter: off; limiter: off).

### Acoustic analyses

Soundscape recordings were analysed by the same observer who surveyed birds in the field (MJ or PS). The observer analysed recordings only from recording points at which they surveyed birds in the field, two months after the end of fieldwork. Observers did not have opportunity to check the list of bird species detected during point-count field survey before scanning the recording from that survey. This way we kept the observer skills consistent at a recording site and eliminated potential effect of previous experience from field survey on results of a recording scanning. The observers listened to recordings and manually scanned spectrograms using Audacity 2.3.2 software, without limit of time spend on analysing a single recording. Each time they analysed a 10-minute recording sample collected exactly at the same time as a point-count done by human-observer in the field. For each recording, a list of detected bird species was prepared. Observers focused on both songs and calls produced by individuals to recognise a species. Special attention was given to recordings where many species sang at the same time, which made species detection and identification difficult. When the fieldworker had difficulties with recognition of some birds vocalisations, he compared them with the samples of vocalisations available on Xeno-Canto (Xeno-canto foundation). However, we found some sound samples which we were not able to assign to a particular species. These unrecognised sounds were excluded from the analysis.

### Statistical analyses

For each survey at recording site, we calculated the number of species detected by recorder and observer over distance ≤ 50 m, ≤ 100 m, and unlimited range. We applied Generalised Estimating Equations (GEE), which extend the generalized linear model to allow for analysis of repeated measurements or other correlated observations [46]. GEE were used to examine the effect of the method of detection, observer and time in a season on the number of detected bird species. In the model we specified the recording point as the subject, survey (early or late) and method of detection (recorder, observer ≤ 50 m, ≤ 100 m, and within unlimited radius) as within-subject variables, and the number of detected bird species as the dependent variable. Data were fitted by a negative binomial distribution with a log-link function. We conducted main-effect models which contained three factors: method of detection, survey (early or late) and observer (MJ or PS). Four independent models considering (1) all bird biodiversity, (2) songbird biodiversity, (3) meadow bird biodiversity and (4) farmland bird biodiversity were conducted (see S2 Table for list of species included in each analysis).

To test whether detection rates of different species determined by acoustic methods differ from the rates of observers surveying birds within a fixed and unlimited distance categories we chose the most common bird species observed during our study, i.e., those which were recorded during more than 10% of surveys (15 or more surveys from 148 conducted together during early and late surveys). For each of those species we ran Wilcoxon two-related samples test. In these tests we compared detection rate by recorder with detection rate by observer surveying birds within 50 m, 100 m, and unlimited distance. We defined the detection rate as a chance to detect the species present at recording site during a single survey by each of the methods. A species was considered present if it was detected during a single, 10-minute survey by at least one of the methods. In this analysis we considered early and late survey at recording site as independent measurements. We applied simple Bonferroni correction for multiple comparisons ($\bar{\alpha }=\frac{\alpha }{n}$, where n is the number of tests). All statistical analyses were conducted in IBM SPSS Statistics 26. P-values are two-tailed.

### Ethics statement

Our study did not involve experiments with animals, therefore we did not need any special permissions. Access to our study area was not restricted in any way. According with Polish law, access to public and private-owners lands is not restricted, excluding areas strictly designated as no entry. We did not encounter areas to which accessibility was limited or illicit. No specific permissions were required for conducting our study.

## Results

### Bird biodiversity estimation by recorder and human-observer

**Fig 1:**
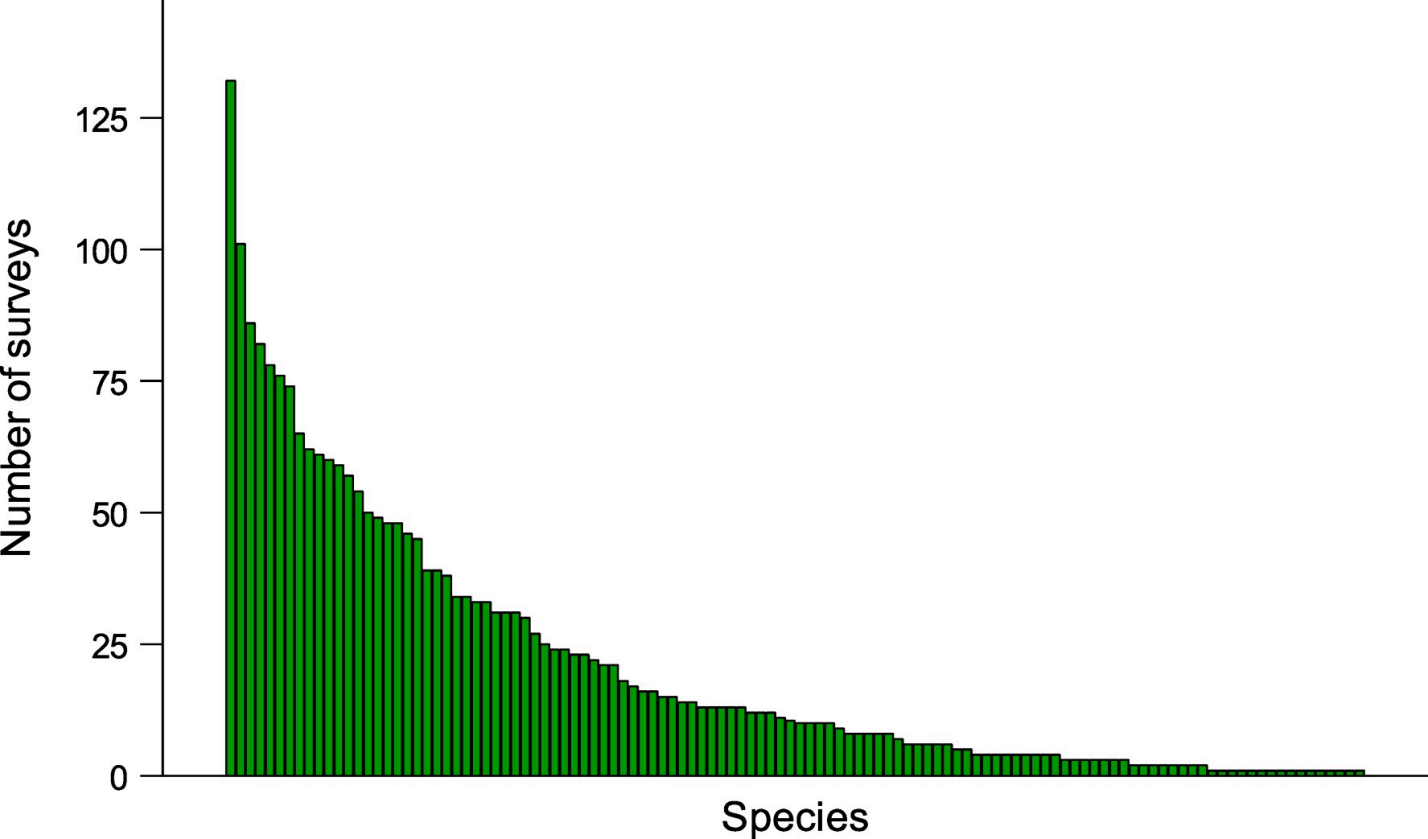
Number of surveys during which particular bird species were detected, independently of the detection method (recorder, observer or both). Graph based on 148 surveys. The most widespread species, European skylark was detected during 132 surveys.

During the study we recorded 117 bird species in total, including 64 songbird species, 17 farmland species, and 15 meadow species (S3 Table). 106 species were recorded during the early survey and 94 species during the late survey. 17 species were observed in more than 50% recording points. The most widespread species, the Eurasian skylark *Alauda arvensis*, was recorded in 92% of recording points. 17 species were recorded at only a single recording point. Seven species were recorded during more than 50% surveys, while 17 species during a single survey. For more details about species distribution and frequency of occurrence see Fig 1 and S3 Table.

**Fig 2:**
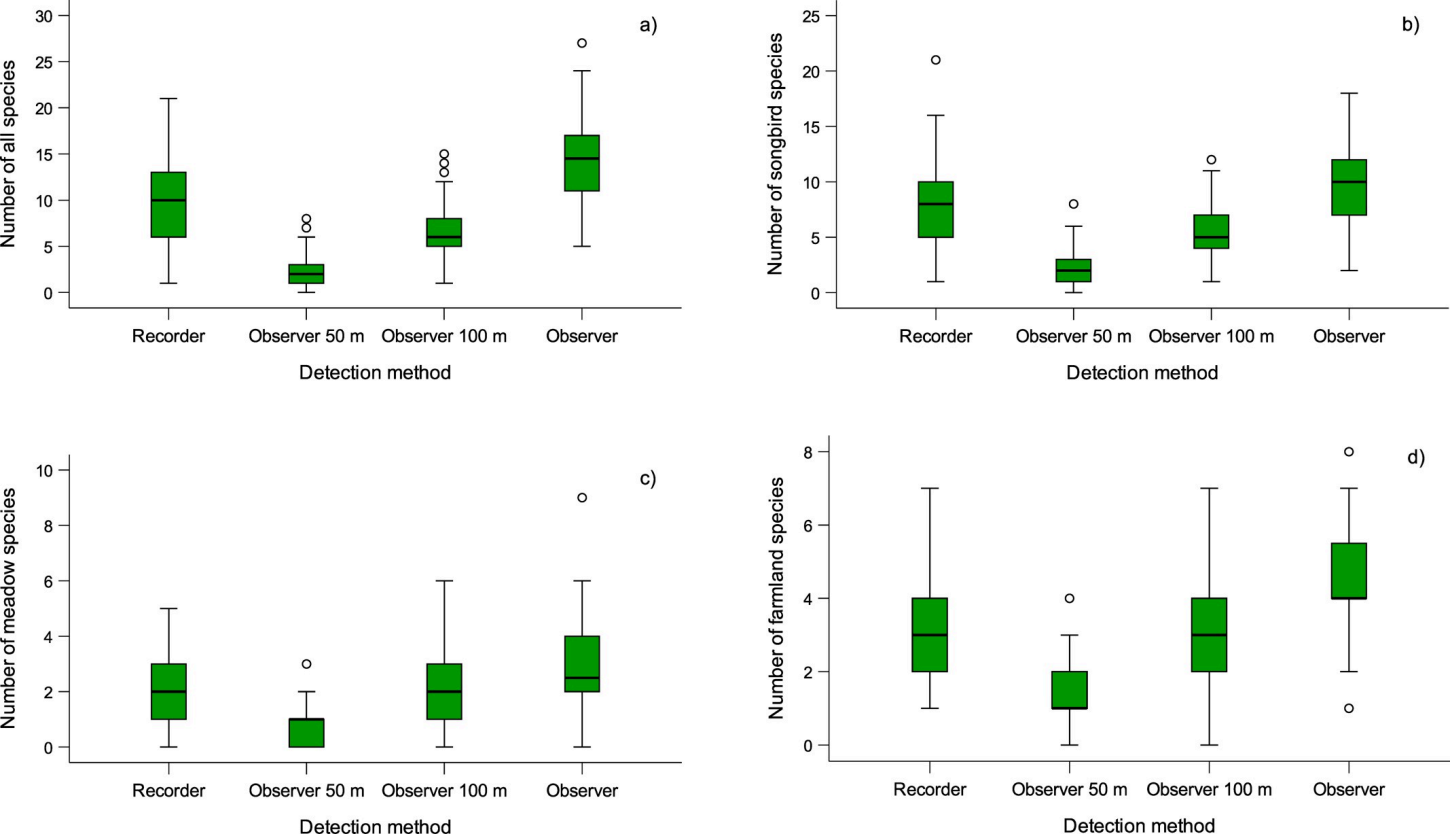
Number of detected bird species by various detection methods. Graphs show (a) all bird species, (b) songbird species, (c) farmland bird species and (d) meadow bird species by four detection methods: (1) recorder, (2) observer surveying birds within 50 m radius, (3) observer surveying birds within 100 m radius, and (4) observer surveying birds in unlimited radius. Boxplots show the median, interquartile range, and outliers.

We found a significant effect of detection method (χ^2^ = 1043.124; p<0 .001; df = 3), observer (χ^2^ = 18.248; p<0.001; df = 1), and no effect of time in a season (χ^2^ = 0.420; p = 0.517; df = 1) on the number of detected bird species. The recorder method detected (1) significantly more species (on average 9.84 species) than the observer up to 50 m (on average 2.22 species) and (2) observer up to 100 m (on average 6.60 species) but (3) significantly fewer species than the observer within an unlimited radius (on average 14.42 species) (Fig 2, Table 1). When we considered only songbird species, we found a significant effect of detection method (χ^2^ = 674.070; p<0.001; df = 3), observer (χ^2^ = 10.622; p<0.001; df = 1) and no effect of time in a season (χ^2^ = 1.409; p = 0.235; df = 1) on the number of detected species. The recorder method detected (1) significantly more species (on average 7.67 species) than the observer up to 50 m (on average 1.99 species) and (2) observer up to 100 m (on average 5.39 species) but (3) significantly fewer species than observer without distance limitation (on average 9.80 species). In the case of farmland bird species, we found a significant effect of detection method (χ^2^ = 501.075; p<0.001; df = 3), observer (χ^2^ = 7.774; p = 0.005; df = 1) and time in the season (χ^2^ = 8.701; p = 0.003; df = 1) on the number of detected species. The recorder method detected (1) significantly more species (on average 2.90 species) than the observer up to 50 m (on average 1.20 species), (2) significantly fewer species than observer without distance limitation (on average 4.50 species) and (3) a statistically indistinguishable number of species from observer up to 100 m (on average 2.98 species). In the case of meadow bird species, we found a significant effect of detection method (χ^2^ = 271.605; p<0.001; df = 3) and no effect of observer (χ^2^ = 0.137; p = 0.712; df = 1) and time in the season (χ^2^ = 0.024; p = 0.877; df = 1). The recorder detected (1) significantly more species (on average 1.86 species) than the observer up to 50 m (on average 0.68 species), (2) significantly fewer species than the observer without distance limitation (on average 2.67 species) and (3) a statistically indistinguishable number of species from the observer up to 100 m (on average 1.84 species).

**Table 1:** Results of three generalised estimating equations.

	All bird species				
	B	SE	Wald χ2	df	p
(Intercept)	2.468	0.0602	1681.515	1	<0.001
Method [observer unlimited]	0.388	0.0266	212.675	1	<0.001
Method [observer 100 m]	-0.400	0.0405	97.414	1	<0.001
Method [observer 50 m]	-1.492	0.0717	433.160	1	<0.001
Survey [late]	-0.023	0.0350	0.420	1	0.517
Observer ID [2]	-0.284	0.0665	18.248	1	<0.001
	Songbird species				
	B	SE	Wald χ2	df	p
(Intercept)	2.154	0.0673	1024.928	1	<0.001
Method [observer unlimited]	0.252	0.026	94.254	1	<0.001
Method [observer 100 m]	-0.349	0.0418	69.703	1	<0.001
Method [observer 50 m]	-1.351	0.0729	343.587	1	<0.001
Survey [late]	0.049	0.0413	1.409	1	<0.235
Observer ID [2]	-0.237	0.0727	10/633	1	<0.001
	Farmland bird species				
	B	SE	Wald χ2	df	p
(Intercept)	1.100	0.0648	288.471	1	<0.001
Method [observer unlimited]	0.444	0.0356	156.083	1	<0.001
Method [observer 100 m]	0.030	0.0414	0.519	1	0.471
Method [observer 50 m]	-0.883	0.0656	181.295	1	<0.001
Survey [late]	0.145	0.0491	8.701	1	0.003
Observer ID [2]	-0.182	0.0652	7.774	1	0.005
	Meadow bird species				
	B	SE	Wald χ2	df	p
(Intercept)	0.588	0.1126	27.311	1	<0.001
Method [observer unlimited]	0.360	0.0509	49.851	1	<0.001
Method [observer 100 m]	-0.010	0.0530	0.034	1	0.854
Method [observer 50 m]	-1.002	0.0963	108.374	1	<0.001
Survey [late]	0.011	0.0730	0.024	1	0.877
Observer ID [2]	0.043	0.1177	0.137	1	0.712

Models examining the effect of detection method (recorder, observer up to 50 m, observer up to 100 m, observer surveying birds in unlimited distance), time of survey (early or late) and observer (MJ or PS) on the number of detected bird species. Separate models were conducted for: (1) all bird species, (2) songbird species, (3) farmland bird species, and (4) meadow bird species. As a reference category we used: recorder for Detection method, early survey for Survey and first observer for Observer ID.

### Detection rate of common birds

During the study 20 bird species were only detected by the human-observer method (three songbirds, two meadow and two farmland bird species; in total 63 detections, from 1 to 13 per species), while five species were only detected by the recorder method (three songbirds, no farmland or meadow species; in total 7 detections, from one to three per species; see S3 and S4 Tables). From the 48 most common bird species that were analysed, we found a statistically indistinguishable difference in the detection rate by recorder and: (1) observer surveying birds within 50 m radius in the case of 12 species, (2) observer surveying birds within 100 m radius in the case of 22 species, and (3) observer surveying birds in unlimited radius in the case of 27 species (Table 2). The detection rate by a recorder in comparison with a human-observer surveying birds was: (1) significantly higher in the case of 36 species and was not lower for any species when 50 m radius was considered; (2) was significantly higher in the case of 23 species and lower in the case of three species when 100 m radius was considered; (3) significantly higher in the case of one species (Black-headed gull *Chroicocephalus ridibundus*) and lower in the case of 20 species when unlimited radius was considered. For more details see Table 2 and S1 Fig.

**Table 2:** Results of Wilcoxon two-related samples tests examining differences in detection rate of the most common bird species by recorders and observers surveying birds within 50 m (Obs. 50 m), 100 m (Obs. 100 m) and unlimited radius (Obs. unlimited).

Species (English name)	Species (Latin name)	N	Obs. 50 m	Obs. 100 m	Obs. unlimited
Eurasian skylark	*Alauda arvensis*	132	↓	↓	↔
Yellowhammer	*Emberiza citrinella*	101	↓	↓	↔
Common quail	*Coturnix coturnix*	88	↓	↓	↔
Common wood pigeon	*Columba palumbus*	86	↓	↔	↑
Common starling	*Sturnus vulgaris*	82	↔	↑	↑
Common cuckoo	*Cuculus canorus*	78	↓	↓	↑
Chaffinch	*Fringilla coelebs*	76	↓	↓	↔
Common crane	*Grus grus*	74	↓	↓	↑
Western jackdaw	*Coloeus monedula*	68	↓	↔	↔
Common whitethroat	*Curruca communis*	65	↓	↔	↔
Common blackbird	*Turdus merula*	62	↓	↓	↑
Eurasian blackcap	*Sylvia atricapilla*	61	↓	↓	↔
Common reed bunting	*Emberiza schoeniclus*	59	↔	↔	↑
Sedge warbler	*Acrocephalus schoenobaenus*	57	↓	↓	↔
Hooded crow	*Corvus cornix*	54	↓	↓	↑
Barn swallow	*Hirundo rustica*	50	↔	↑	↑
Thrush nightingale	*Luscinia luscinia*	49	↓	↓	↔
Golden oriole	*Oriolus oriolus*	48	↓	↓	↔
Common pheasant	*Phasianus colchicus*	48	↓	↓	↔
Whinchat	*Saxicola rubetra*	46	↓	↑	↑
White stork	*Ciconia ciconia*	45	↔	↔	↑
Meadow pipit	*Anthus pratensis*	39	↓	↔	↑
Song thrush	*Turdus philomelos*	38	↓	↓	↔
Willow warbler	*Phylloscopus trochilus*	37	↓	↓	↔
Black-headed gull	*Chroicocephalus ridibundus*	35	↓	↔	↓
Common raven	*Corvus corax*	34	↓	↓	↑
Fieldfare	*Turdus pilaris*	33	↓	↔	↔
Common buzzard	*Buteo buteo*	33	↓	↔	↑
Grasshopper warbler	*Locustella naevia*	31	↓	↓	↔
Red-backed shrike	*Lanius collurio*	31	↔	↔	↑
Northern lapwing	*Vanellus vanellus*	31	↓	↔	↑
Common snipe	*Gallinago gallinago*	30	↓	↔	↑
Eurasian magpie	*Pica pica*	27	↓	↓	↔
Great tit	*Parus major*	25	↓	↔	↔
Western yellow wagtail	*Motacilla flava*	24	↓	↔	↔
Common chiffchaff	*Phylloscopus collybita*	24	↓	↓	↔
Common rosefinch	*Carpodacus erythrinus*	24	↓	↓	↔
European goldfinch	*Carduelis carduelis*	23	↓	↔	↔
Eurasian hoopoe	*Upupa epops*	23	↔	↓	↑
Mallard	*Anas platyrhynchos*	22	↔	↔	↑
Great reed warbler	*Acrocephalus arundinaceus*	21	↓	↓	↔
Marsh warbler	*Acrocephalus palustris*	21	↔	↔	↑
Eurasian collared dove	*Streptopelia decaocto*	18	↓	↓	↔
Sand martin	*Riparia riparia*	18	↔	↔	↔
Western marsh harrier	*Circus aeruginosus*	16	↔	↔	↑
Corn bunting	*Emberiza calandra*	16	↓	↔	↔
Great-spotted woodpecker	*Dendrocopos major*	15	↔	↔	↔
Eurasian blue tit	*Cyanistes caeruleus*	15	↔	↔	↔
Summary		↔	12	22	27
		↓	0	3	20
		↑	36	23	1

↓–detection rate is significantly lower in comparison to the detection by recorder; ↑–detection rate is significantly higher in comparison to the detection by recorder; ↔–no statistical significant difference in detection rate in comparison to detection by recorder. Bonferroni correction for multiple comparisons was applied ($\bar{\alpha }=\frac{\alpha }{n}$, where n is the number of tests). N–number of surveys during which species was detected at recording site independently of the method of detection. For exact results of Wilcoxon two-related samples tests see S1 Fig.

## Discussion

### Bird biodiversity estimation in unlimited detection radius

The results of our study show that in open habitats an acoustic recorder detects fewer bird species compared to a highly skilled observer when unlimited radius is applied by the human-observer and more species when the observer surveys birds within a fixed radius (50 m or 100 m in our case). The difference between the methods may arise from visual detections of additional species during the field survey by the observer [47] and due to the various areas sampled by recorder and observer [20,30]. Open habitats should strongly support visual detections by observers [23]. Thus, birds that are at a great distance from a survey point, or species which are silent for the most time (e.g., storks, herons, birds of prey), should be detected by a human-observer visually and undetected on the recorder, because they do not vocalise, or their vocalisation is too low to be recorded. Additionally, some birds avoid the surrounding area of the point after the arrival of an observer [48], potentially leading to a decrease in the probability of detection of birds near to a survey point.

Unlimited detection radius has been applied in many studies comparing the effectiveness of acoustic recorders and human-observers in bird biodiversity estimation. Most of them reported higher biodiversity detected by human-observers than by recorders [22,47], which is an expected result. In general, recorders have similar or lower detection range than human-observers [32]. Therefore, with no distance limitation, experienced human-observers should detect more or the same number of species as recorders, because both techniques detect the same vocalising species, whereas observers have the additional opportunity to detect vocalising species from a further distance and all silent species that are within visibility range of the observer [32]. Moreover, difference in the number of species detected by recorder and observer should be greater in open than in closed habitats, since that chance for visual detections only is higher in the former [23]. A lower number of species detected by human-observer than recorder within the unlimited distance suggests that the observer has overlooked some vocalising species during the field survey. The proportion of overlooked detections by observer in the field to the total number of species detections recorded by recorder can be used as a measure of the skills of observer. This way studies involving observers of varying skills could be compared.

To effectively accomplish the monitoring of bird biodiversity, it is important to survey birds in the same locations, using the same method for many years [45]. Acoustic monitoring without distance limitation fulfils these assumptions. Data are collected in a highly standardised way: the same equipment and recording quality, constant acoustic properties of environment at particular recording sites, constant within-species detection range (but diverse between-species). In this way the acoustic monitoring generates a presence-absence indicator, which enables the tracking of environmental changes in a simple and reliable way, like in Aquatic warbler *Acrocephalus paludicola*, in which presence and abundance of singing males allows to rank the habitat quality and its changes [49]. Another issue is whether certain species are overlooked using acoustic recorders when conducting the biodiversity monitoring. In our study we found 20 species that were undetected by the recorder (S4 Table). Seven of them were birds of prey, which have broad territories and, excluding Montagu’s harrier, breed outside the meadows. Another six species do not breed in meadows (herons, terns, cormorants, crossbills), five of them were only observed flying over the study area. Two other species, undetected by the recorder, (Ruff sandpiper *Calidris pugnax* and Green sandpiper *Tringa ochropus*) were typical spring migrants. Thus, the recorders only missed five species (in total 10 detections: European serin *Serinus serinus*, Partridge *Perdix perdix*, Stock dove *Columba oenas*, Turtle dove *Streptopelia turtur*, River warbler *Locustella fluviatilis*) that breed in meadows and are important from the perspective of meadow bird biodiversity. On the other hand, field observers did not detect five species whose vocalisations were recorded by the recorder. However, it was just seven detections belonging to five species (S3 and S4 Tables). None of them breeds in the meadows in Poland.

### Bird biodiversity estimation within a fixed detection radius

The effectiveness of bird species detection by a recorder in comparison with human-observer surveying birds within a fixed radius has been examined previously [30,32]. In such approach the distance to a bird is estimated by the observer, while the recorder detects birds without any distance limitation. Our study showed that after applying a fixed detection radius by the human-observer (50 m or 100 m) the recorder detected significantly more bird species than the observer. It means that the effective range of detection by a recorder for most bird species exceeds 100 m (Table 1, Figs 1 and S1). Looking at a single species (Table 2, S1 Fig) we can see that detection rate for most of them is higher by recorder than by human-observer surveying birds within 50 m and 100 m distance (36 and 23 species, respectively), while in unlimited distance the most species are detected similarly by observer and recorder.

The visual and audio detection rate of a species depends on their behaviour and call characteristics. In general, species that are small, hidden or difficult to visually recognise (e.g., Common whitethroat *Curruca communis*, Common quail *Coturnix coturnix*, Marsh warbler *Acrocephalus palustris*) are detected similarly by recorder and observer from 100 m or unlimited distance, dependently on the loudness of their vocalisation, while detection rate of easily seen species including small (e.g., Whinchat *Saxicola rubetra*) and large ones (e.g, White stork *Ciconia ciconia* or Common crane *Grus gus*), is higher for human-observers within unlimited distance than for recorders. However, it is possible to find species (Table 2) or groups of species (e.g., meadow or farmland species; Table 1, Fig 1) that are detected with a similar detection rate within a 100 m radius both for the recorder and human-observer. Furthermore, the overall bird species richness is not necessarily the best indicator when monitoring is conducted in a very specific environment [43]. Therefore, we examined the effectiveness of acoustic recorders in the estimation of meadow and farmland bird biodiversity–two groups of birds specific to meadow habitat and important from the perspective of monitoring of agricultural landscape. In both groups we found that the recorder and human-observer surveying birds within a 100 m radius provide similar estimations of bird biodiversity. Therefore, the traditional observer-based monitoring of these two groups of birds can be successfully substituted with acoustic monitoring at least in this regional habitat, even without any corrections for acoustic properties of environment, habitat type and detection distance of a particular species.

### Applying acoustic monitoring of meadow species in practice

Our results allow to propose acoustic monitoring as equally accurate and potentially more efficient alternative to traditional field surveys conducted by ornithologists in agricultural landscapes. The first advantage of acoustic monitoring is that data can be collected during a short breeding season in the field by non-specialists (e.g., local agriculture management authorities, landowners) in very standardized way (time during a season and day, survey duration, weather conditions, equipment), and analysed later in the lab. Therefore, acoustic monitoring enables us to sample incomparably more locations than by using traditional observer-based approach. The second advantage is standardised acoustic data analysis. In our approach we applied the simplest method–manual spectrogram scanning and listening to recordings. Even using this method, collected during the short breeding season data can be analysed in the rest part of the year. However, applying acoustic monitoring at regional or national level enables the use of automatic species detection and recognition algorithms [50,51] or applying acoustic indicators [52], which is more efficient and more acceptable for incorporation into big data analysis. Moreover, soundscape recordings can be used to monitor other vocalising taxa, such as insects or amphibians [7,53], whose importance is increasingly appreciated but the monitoring is still insufficient and difficult to conduct. In the simplest way acoustic monitoring could be, for example, implemented to evaluate the effectiveness of agri-environmental schemes undertaken by many European Union countries to protect bird biodiversity in farmland landscapes or any other protection programs which need to measure the effect of undertaken activities. Such citizen-science approach could involve landowners or volunteers collecting sound samples using digital recorders (e.g., mobile phones) on their parcels before and during the undertaking of the agri-environmental commitment, and then send recordings to the units responsible for management and monitoring of the effectiveness of agri-environmental measures, responsible for data analysis. Applying acoustic monitoring of agriculture landscape is possible through different scenarios. All of them need planning and management at regional or national level, in accordance with current guidance for acoustic monitoring of environment [9–12]. In this study we showed that using acoustic monitoring we can estimate farmland and meadows bird biodiversity just as well as when using traditional human-based approach.

The advantage of acoustic monitoring is that data are collected and analysed in standardised way, the costs of acoustic monitoring are lower in comparison to traditional human-based approach [10], and it is possible to increase survey duration to get more accurate and precise estimation of population parameters [23].

## Conclusions

Our study showed that:

- Highly skilled human observers surveying birds within unlimited radius detect more species than acoustic recorders, because acoustic detection range of observers is longer than recorders and observers may detect visually species which are silent.
- Meadows and farmland bird biodiversity is equally estimated by recorders and human observers surveying birds within 100 m radius. Therefore, it is possible to replace traditional, human based point-counts by acoustic approach without losing effectiveness in detection of the species which are the most common and typical for agriculture landscape.
- Although recorders do not detect some silent and very distant species, acoustic approach can be effectively adapted to long-term, large-scale monitoring of birds in agriculture landscape.

## Supporting information

S1 Fig Detection rate (± SE) of 48 the most common bird species by four detection methods: (1) recorder, (2) observer up to 50 m, (3) observer up to 100 m and (4) observer in unlimited distance. The number of surveys (N) at which the species was detected independently on the method of detection is given. Results of Wilcoxon two-related simple test are given. Tests compare differences in detection rate by recorder and observers surveying birds within 50 m, 100 m and unlimited distance.
(PDF)

S1 Table Summary dataset, containing data from a field survey (soundscape recording and point-counting). Observer, recording site ID, geographical coordinates, survey (early–from April 11 to May 20, hours: 05:08–09:53 AM; late–from May 27 to June 30; hours 05:01–08:59 AM, local time), detection method, number of detected all bird species, songbird species, meadow bird species and farmland bird species, visibility around the recording point are given.
(XLSX)

S2 Table List of bird species defined as meadow and farmland species. (PDF)

S3 Table Occurrence of a particular bird species. Table shows: the number of surveys in which species was observed, the number of recording sites in which species was observed, the number of detections by a recorder, the number of detections by a human-observer.
(XLSX)

S4 Table List of bird species detected only by recorder and only by human-observer. (PDF)
