# Supplementary material for: Acoustic approach as an alternative to human-based survey in bird biodiversity monitoring in agricultural meadows
Source: PLoS One. 2022 Apr 8;17(4):e0266557. doi: 10.1371/journal.pone.0266557 (PMC8992991; doi:10.1371/journal.pone.0266557)
Supplement: S1 Fig — The number of surveys (N) at which the species was detected independently on the method of detection is given. Results of Wilcoxon two-related simple test are given. Tests compare differences in detection rate by recorder and observers surveying birds within 50 m, 100 m and unlimited distance. (PDF) [file pone.0266557.s001.pdf]

S1 Figure. Detection rate (± SE) of 48 the most common bird species by four detection methods: (1) recorder, (2) observer up to 50 m, (3) observer up to 100 m and (4) observer in unlimited distance. The number of surveys (N) at which the species was detected independently on the method of detection is given. Results of Wilcoxon two-related samples tests are given. Tests compare differences in detection rate by recorder (R) and observers surveying birds within 50 m (Ob 50 m), 100 m (Ob 100 m) and unlimited radius (Ob unlm). * - indicates significant difference after Bonferroni correction.

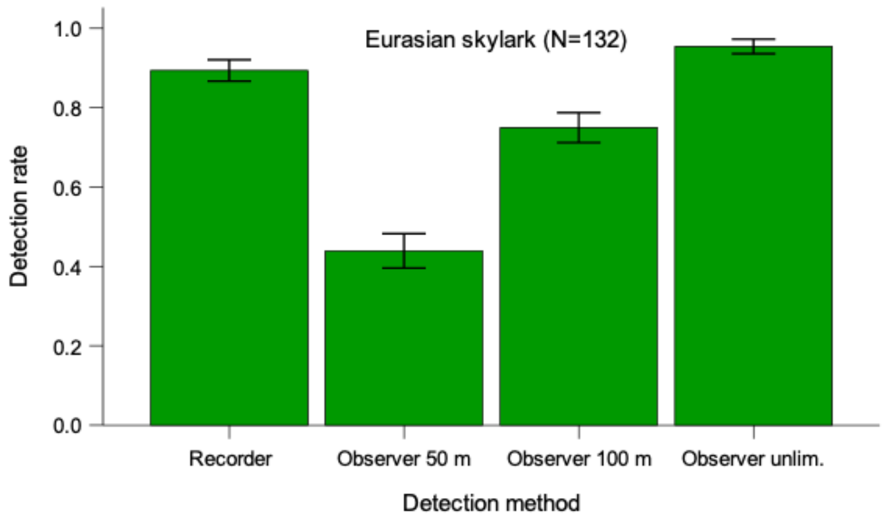

Eurasian skylark

Alauda arvensis

	Z	p
R-Ob 50 m	-7.746	<0.001*
R-Ob 100 m	-3.413	<0.001*
R-Ob unlm	-1.789	0.074

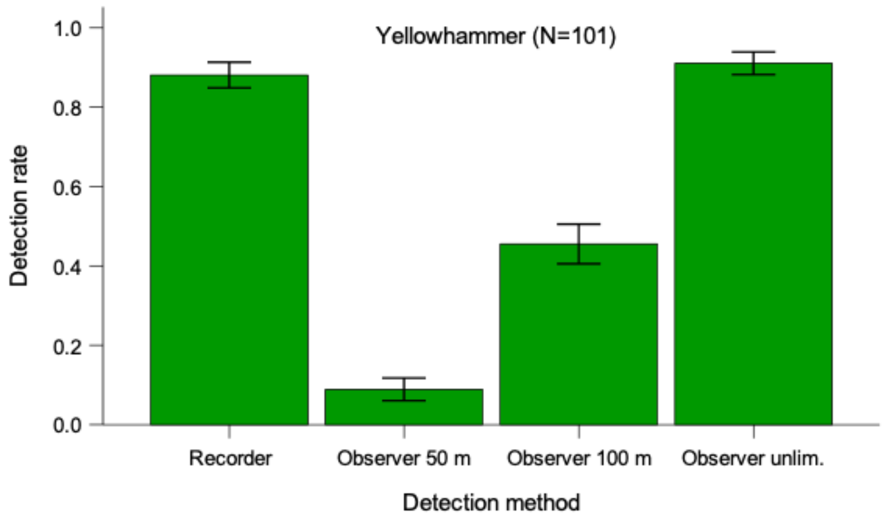

Yellowhammer

Emberiza citrinella

	Z	p
R-Ob 50 m	-8.835	<0.001*
R-Ob 100 m	-6.143	<0.001*
R-Ob unlm	-0.655	0.513

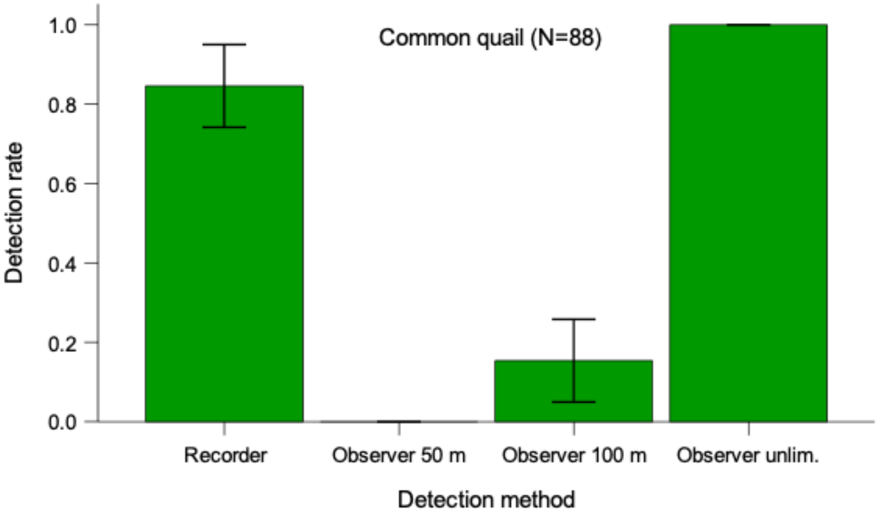

Common quail

Coturnix coturnix

	Z	p
R-Ob 50 m	-3.317	<0.001*
R-Ob 100 m	-3.000	0.003*
R-Ob unlm	-1.414	0.157

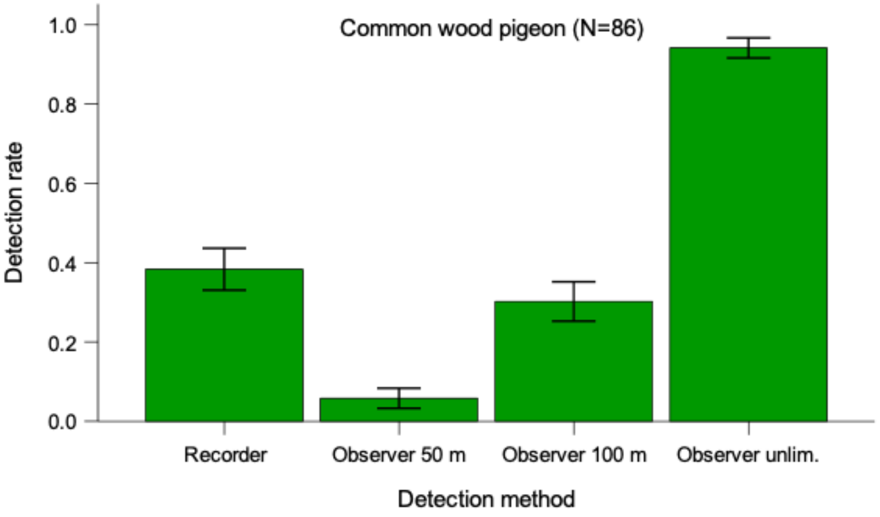

Common wood pigeon

Columba palumbus

	Z	p
R-Ob 50 m	-4.667	<0.001*
R-Ob 100 m	-1.121	0.262
R-Ob unlm	-6.303	<0.001*

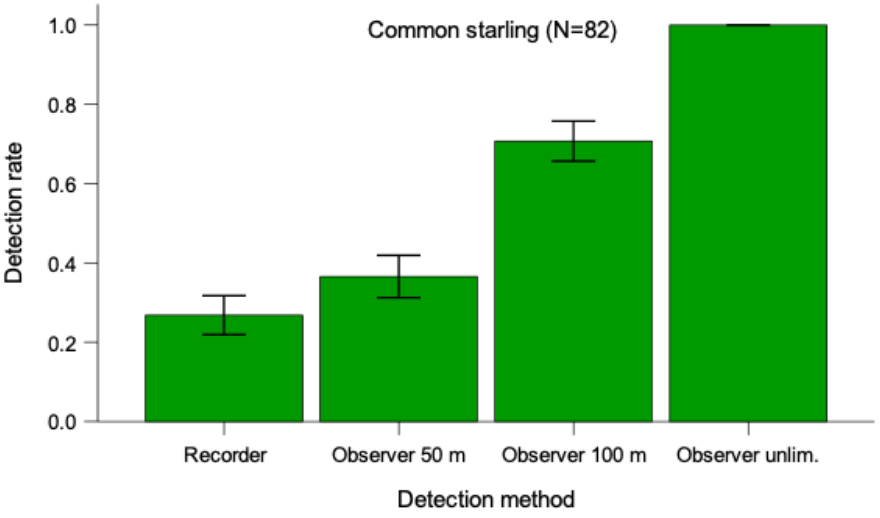

Common starling

Sturnus vulgaris

	Z	p
R-Ob 50 m	-1.512	0.131
R-Ob 100 m	-5.840	<0.001*
R-Ob unlm	-7.746	<0.001*

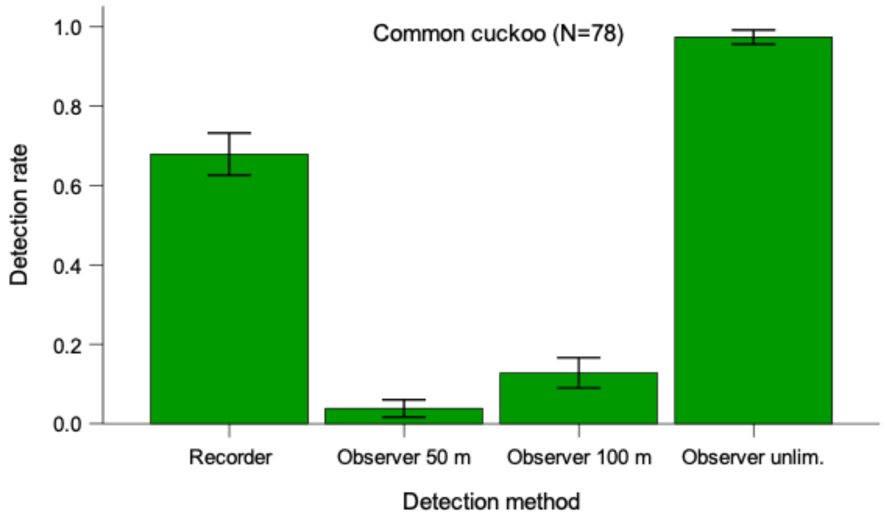

Common cuckoo

Cuculus canorus

	Z	p
R-Ob 50 m	-7.071	<0.001*
R-Ob 100 m	-6.272	<0.001*
R-Ob unlm	-4.426	<0.001*

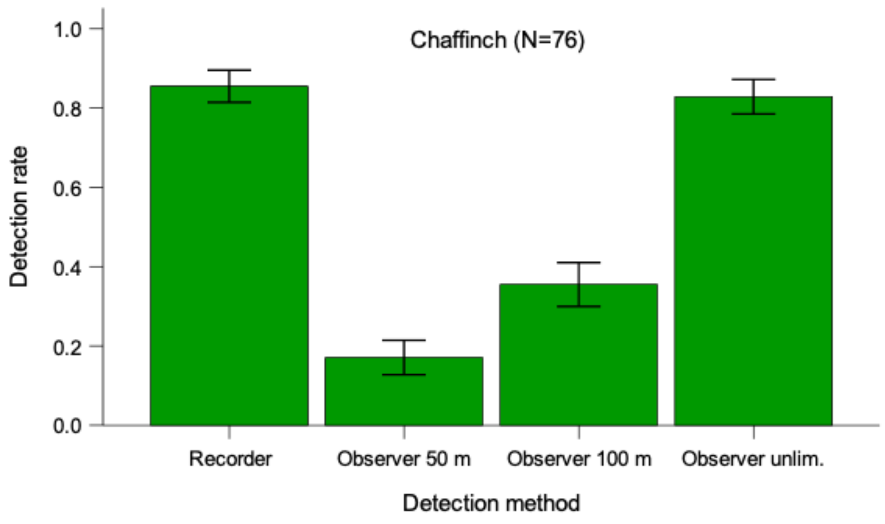

Chaffinch

Fringilla coelebs

	Z	p
R-Ob 50 m	-7.076	<0.001*
R-Ob 100 m	-5.729	<0.001*
R-Ob unlm	-0.408	0.683

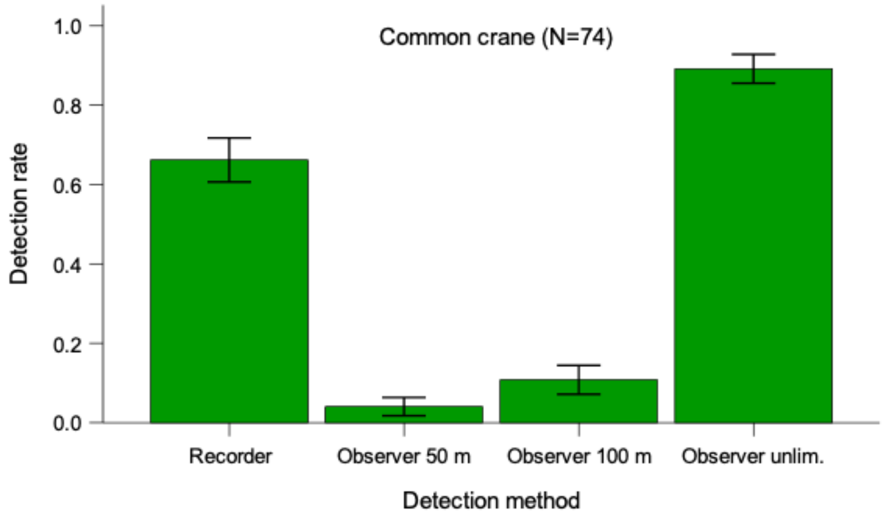

Common crane

Grus grus

	Z	p
R-Ob 50 m	-6.640	<0.001*
R-Ob 100 m	-5.857	<0.001*
R-Ob unlm	-2.959	0.003*

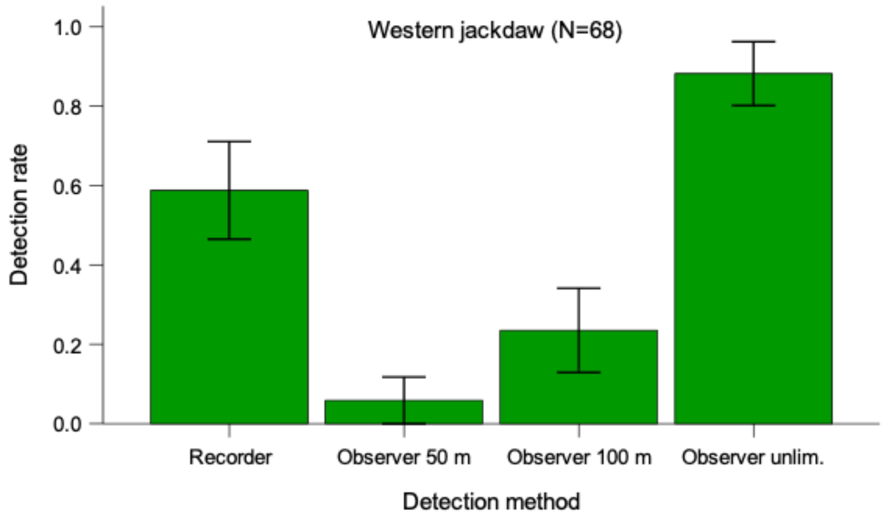

Western jackdaw

Coloeus monedula

	Z	p
R-Ob 50 m	-3.000	0.003*
R-Ob 100 m	-2.121	0.034
R-Ob unlm	-1.667	0.096

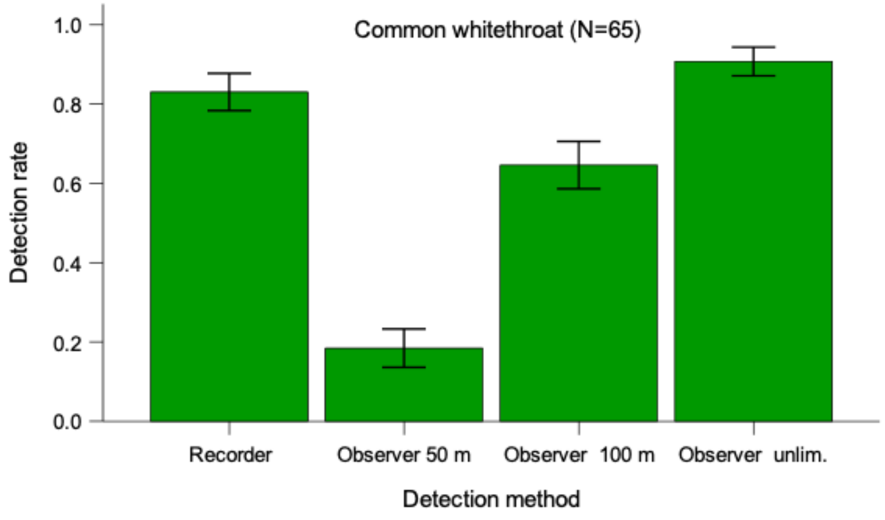

Common whitethroat

Curruca communis

	Z	p
R-Ob 50 m	-6.332	<.000*
R-Ob 100 m	-2.558	0.011
R-Ob unlm	-1.213	0.225

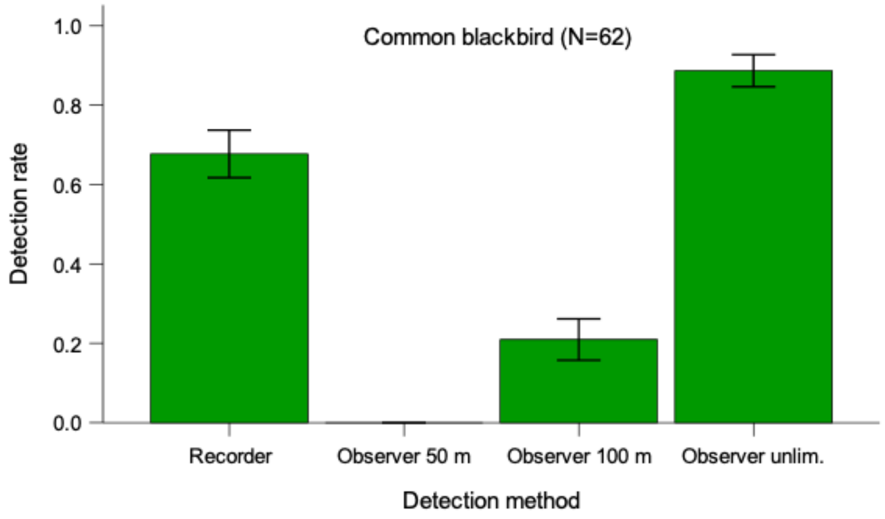

Common blackbird

Turdus merula

	Z	p
R-Ob 50 m	-6.481	<0.001*
R-Ob 100 m	-4.768	<0.001*
R-Ob unlm	-2.502	0.012*

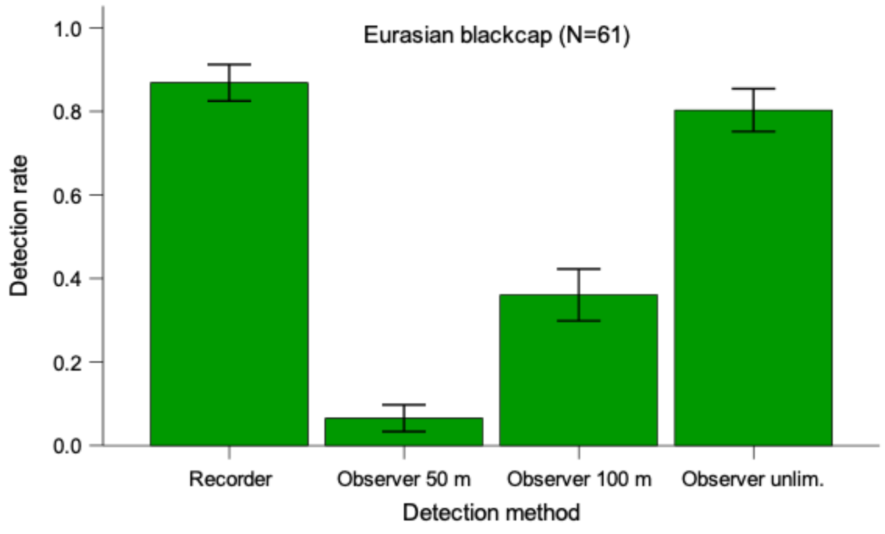

Eurasian blackcap

Sylvia atricapilla

	Z	p
R-Ob 50 m	-7.000	<0.001*
R-Ob 100 m	-5.396	<0.001*
R-Ob unlm	-.894	0.371

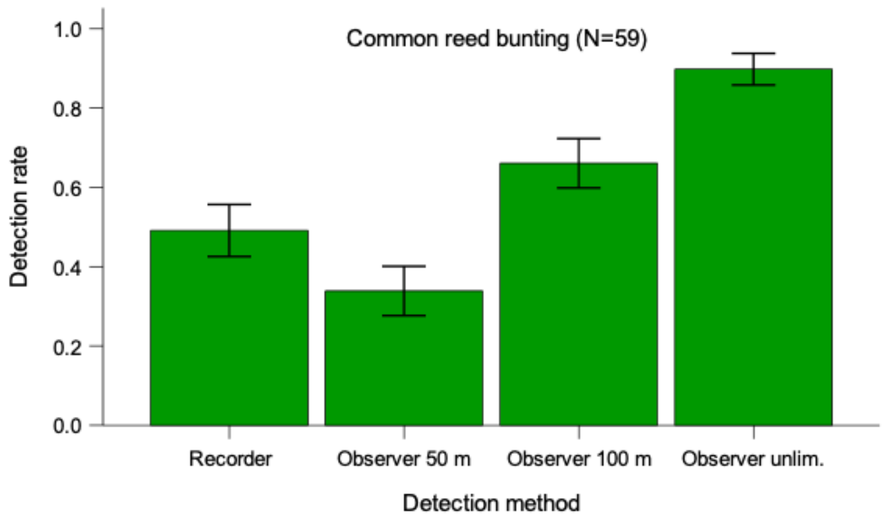

Common reed bunting

Emberiza schoeniclus

	Z	p
R-Ob 50 m	-1.616	0.106
R-Ob 100 m	-1.768	0.077
R-Ob unlm	-4.000	<0.001*

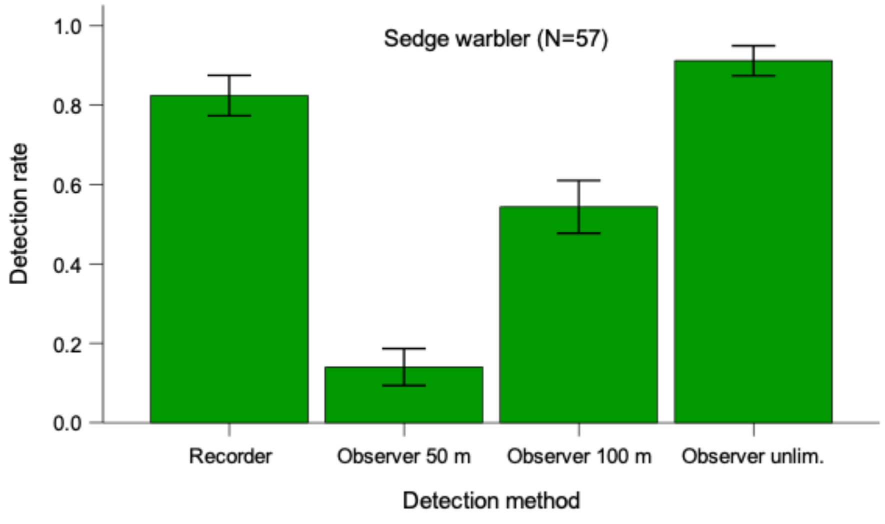

Sedge warbler

Acrocephalus schoenobaenus

	Z	p
R-Ob 50 m	-6.245	<0.001*
R-Ob 100 m	-3.266	<0.001*
R-Ob unlm	-1.291	0.197

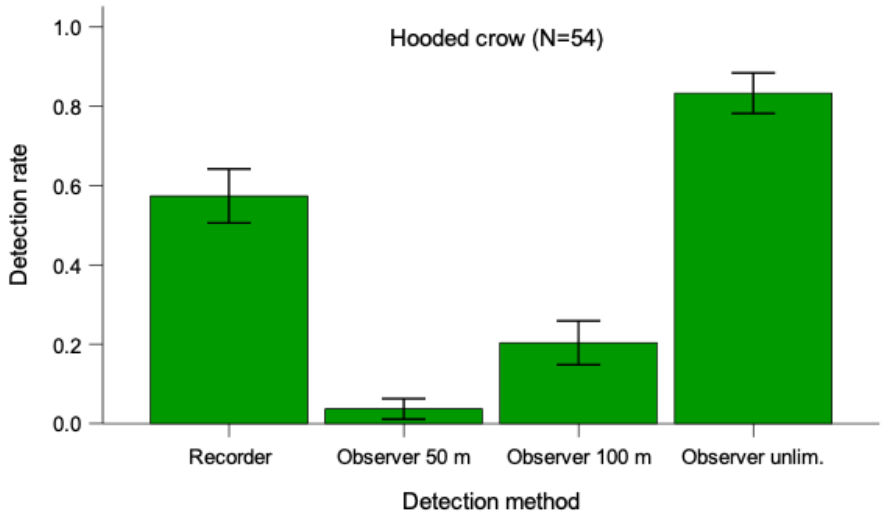

Hooded crow

Corvus cornix

	Z	p
R-Ob 50 m	-5.385	<0.001*
R-Ob 100 m	-4.264	<0.001*
R-Ob unlm	-2.475	0.013*

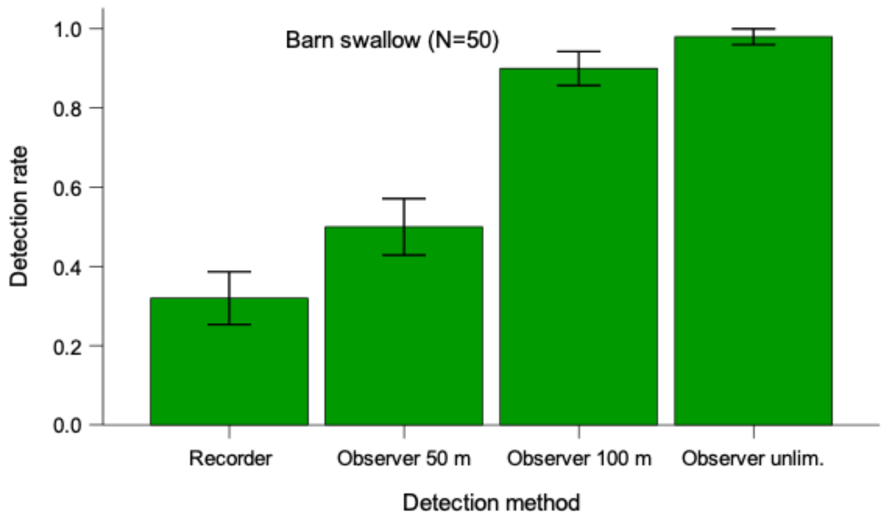

Barn swallow

Hirundo rustica

	Z	p
R-Ob 50 m	-1.964	0.050
R-Ob 100 m	-5.048	<0.001*
R-Ob unlm	-5.578	<0.001*

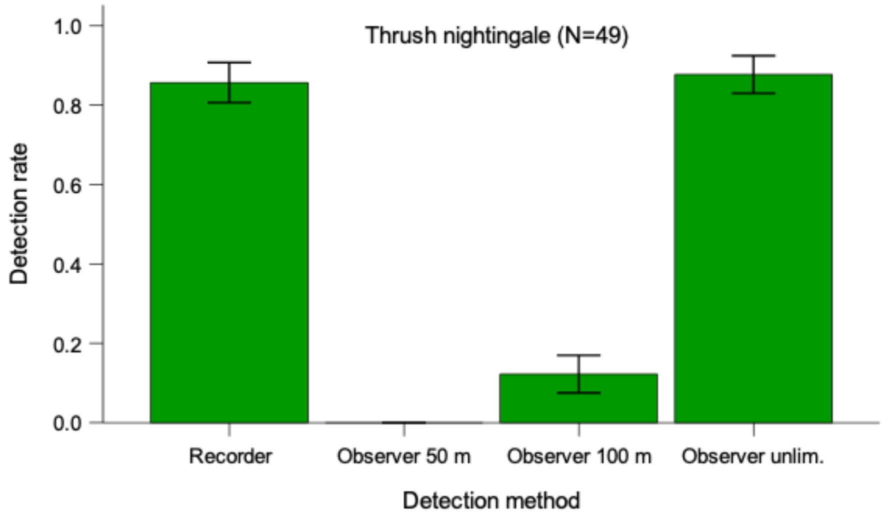

Thrush nightingale

Luscinia luscinia

	Z	p
R-Ob 50 m	-6.481	<0.001*
R-Ob 100 m	-6.000	<0.001*
R-Ob unlm	-0.277	0.782

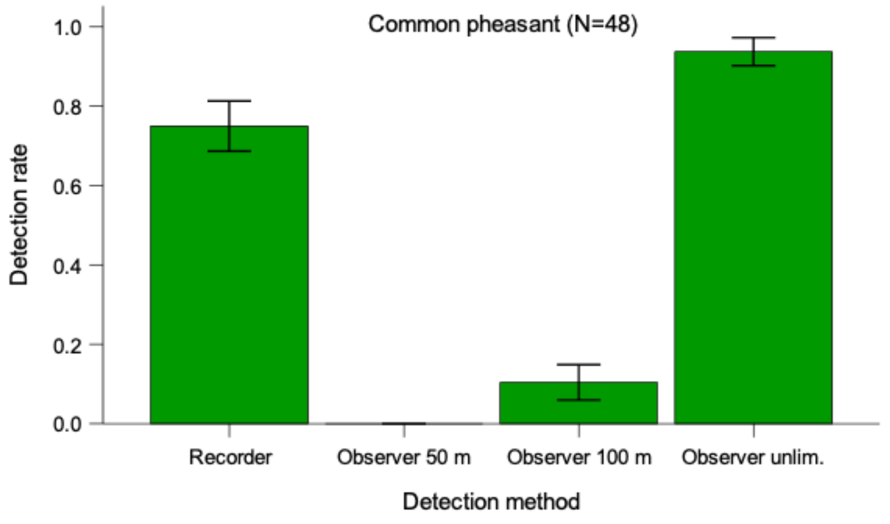

Common pheasant

Phasianus colchicus

	Z	p
R-Ob 50 m	-6.000	<0.001*
R-Ob 100 m	-5.568	<0.001*
R-Ob unlm	-2.324	0.020

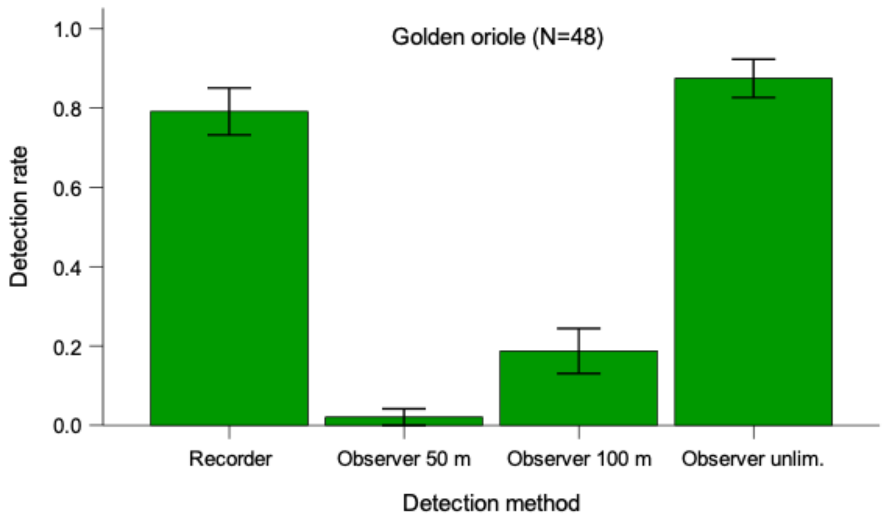

Golden oriole

Oriolus oriolus

	Z	p
R-Ob 50 m	-5.925	<0.001*
R-Ob 100 m	-5.048	<0.001*
R-Ob unlm	-1.000	0.317

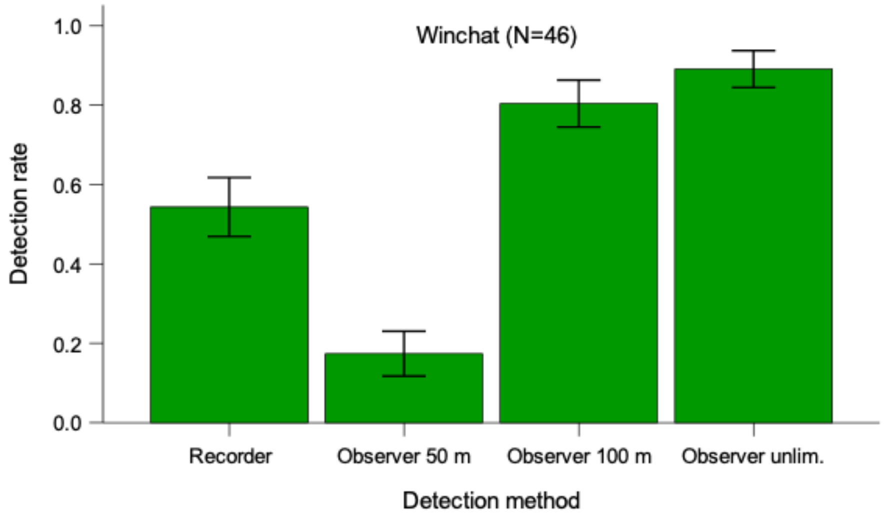

Whinchat

Saxicola rubetra

	Z	p
R-Ob 50 m	-3.157	0.002*
R-Ob 100 m	-2.449	0.014*
R-Ob unlm	-3.138	0.002*

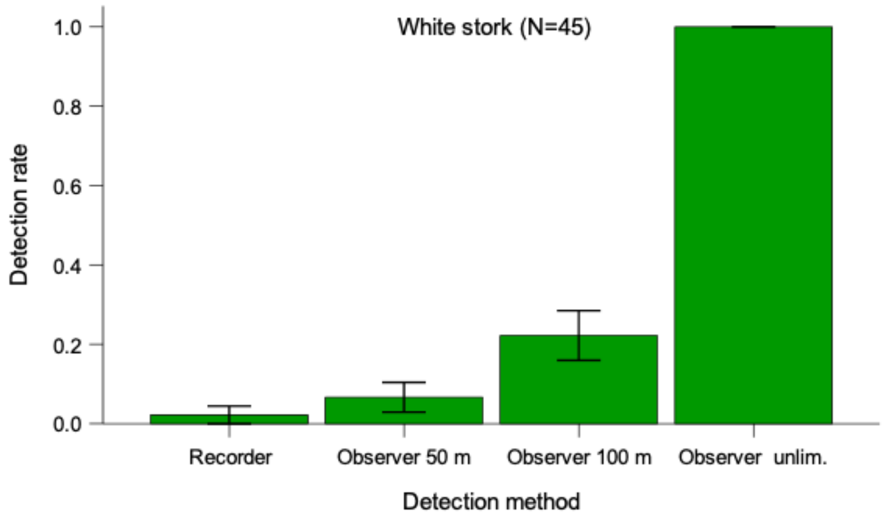

White stork

Ciconia ciconia

	Z	p
R-Ob 50 m	-1.414	0.157
R-Ob 100 m	-1.667	0.096
R-Ob unlm	-2.840	0.005*

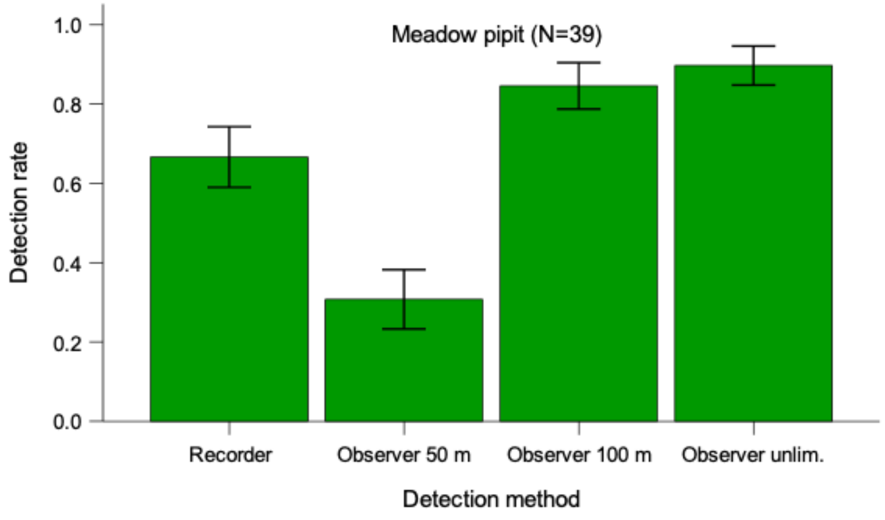

Meadow pipit

Anthus pratensis

	Z	p
R-Ob 50 m	-2.985	0.003*
R-Ob 100 m	-1.698	0.090
R-Ob unlm	-2.183	0.029

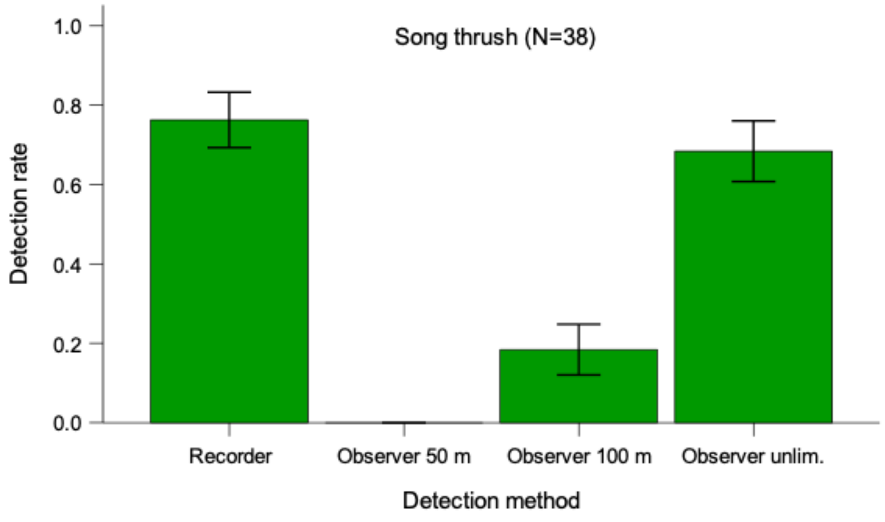

Song thrush

Turdus philomelos

	Z	p
R-Ob 50 m	-5.385	<0.001*
R-Ob 100 m	-4.491	<0.001*
R-Ob unlm	-0.655	0.513

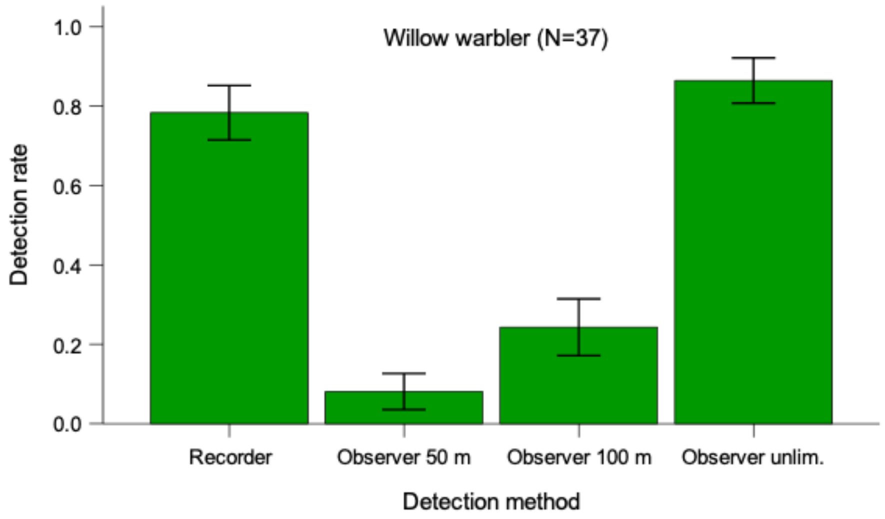

Willow warbler

Phylloscopus trochilus

	Z	p
R-Ob 50 m	-5.099	<0.001*
R-Ob 100 m	-4.472	<0.001*
R-Ob unlm	-0.832	0.405

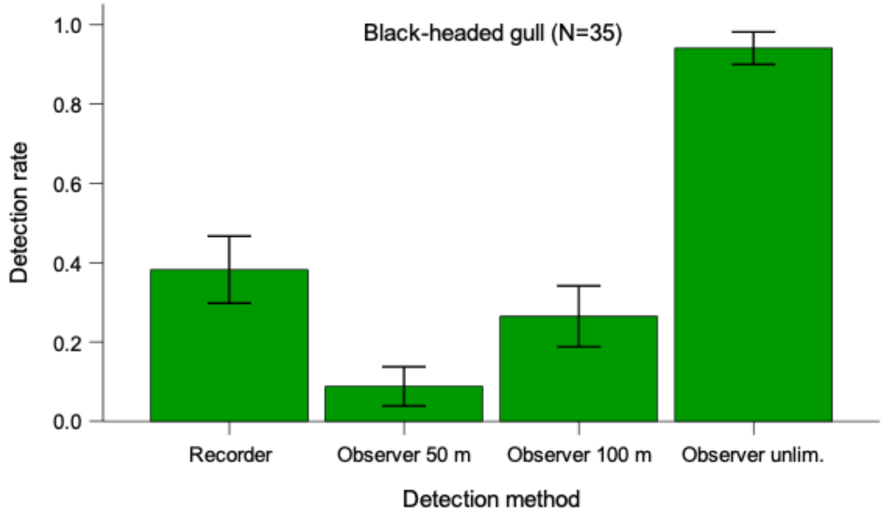

Black-headed gull

Chroicocephalus ridibundus

	Z	p
R-Ob 50 m	-2.887	0.004*
R-Ob 100 m	-1.069	0.285
R-Ob unlm	-3.962	<0.001*

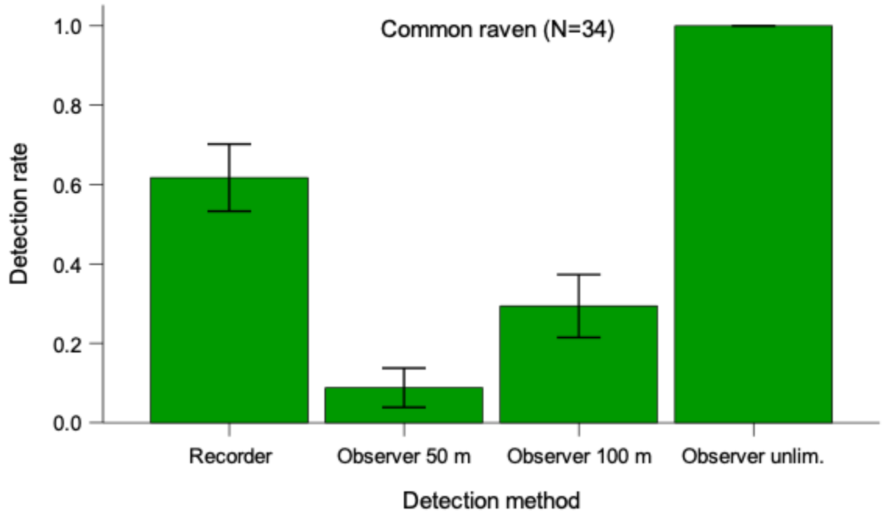

Common raven

Corvus corax

	Z	p
R-Ob 50 m	-4.243	<0.001*
R-Ob 100 m	-2.840	0.005*
R-Ob unlm	-3.606	<0.001*

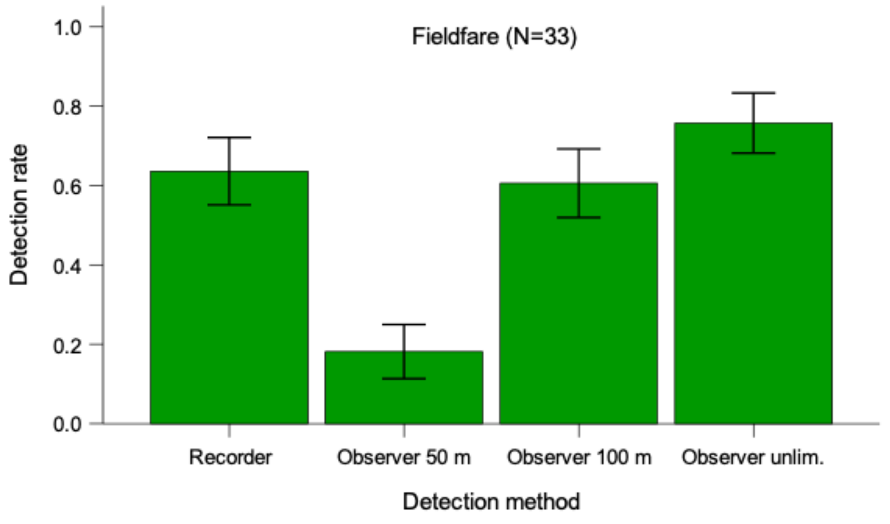

Fieldfare

Turdus pilaris

	Z	p
R-Ob 50 m	-3.638	<0.001*
R-Ob 100 m	-0.243	0.808
R-Ob unlm	-0.894	0.371

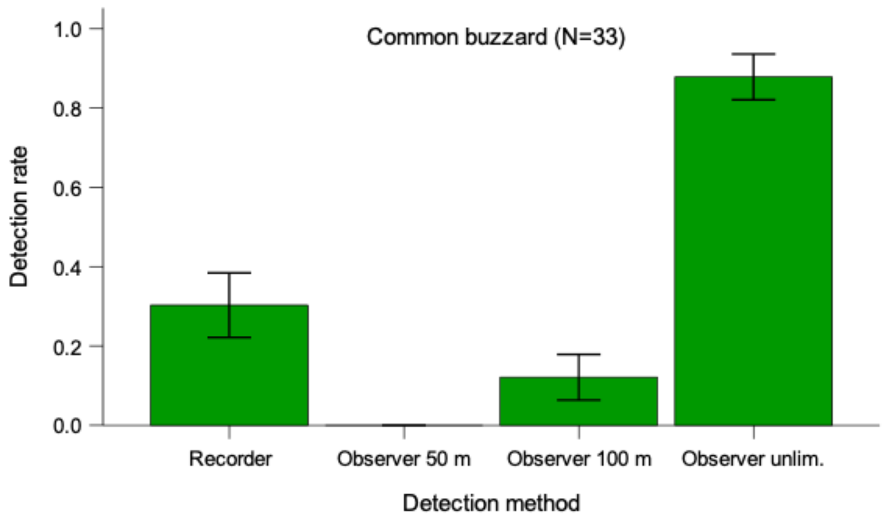

Common buzzard

Buteo buteo

	Z	p
R-Ob 50 m	-3.162	0.002*
R-Ob 100 m	-1.897	0.058
R-Ob unlm	-3.657	0.001*

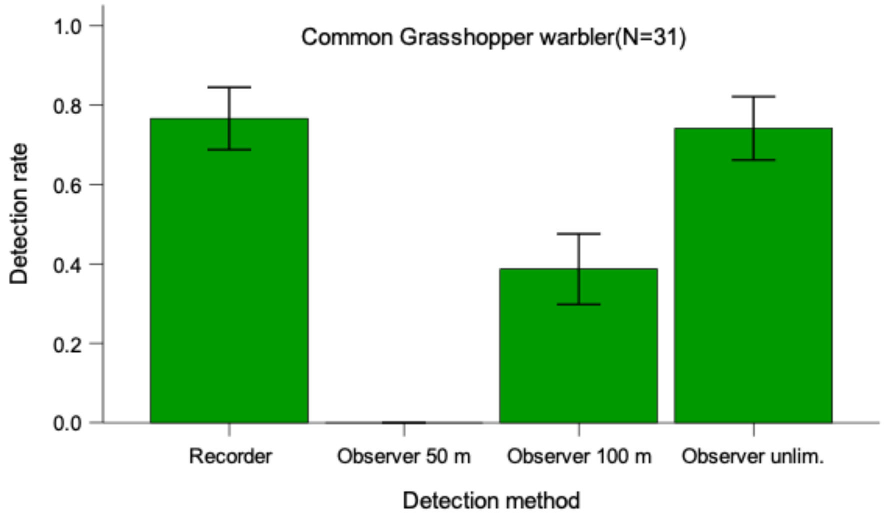

Common grasshopper warbler

Locustella naevia

	Z	p
R-Ob 50 m	-4.796	<0.001*
R-Ob 100 m	-2.524	0.012*
R-Ob unlm	0.001	1.000

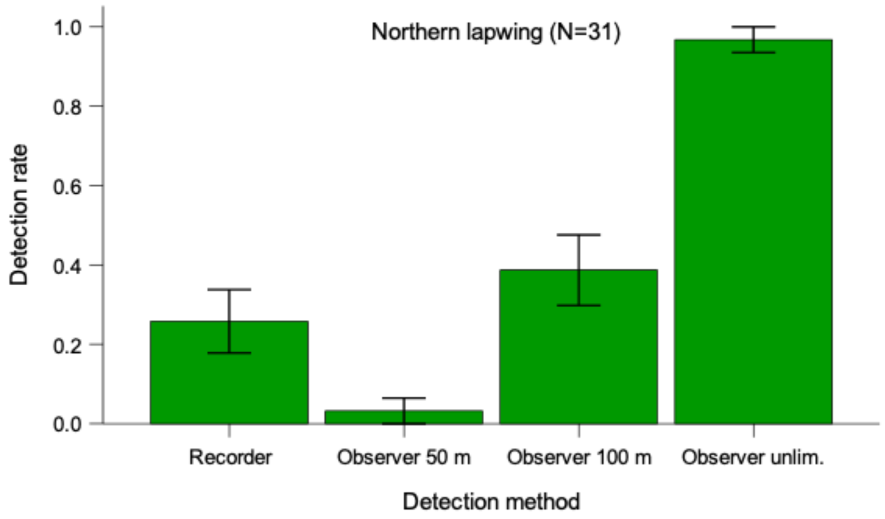

Northern lapwing

Vanellus vanellus

	Z	p
R-Ob 50 m	-2.646	0.008*
R-Ob 100 m	-1.155	0.248
R-Ob unlm	-4.491	<0.001*

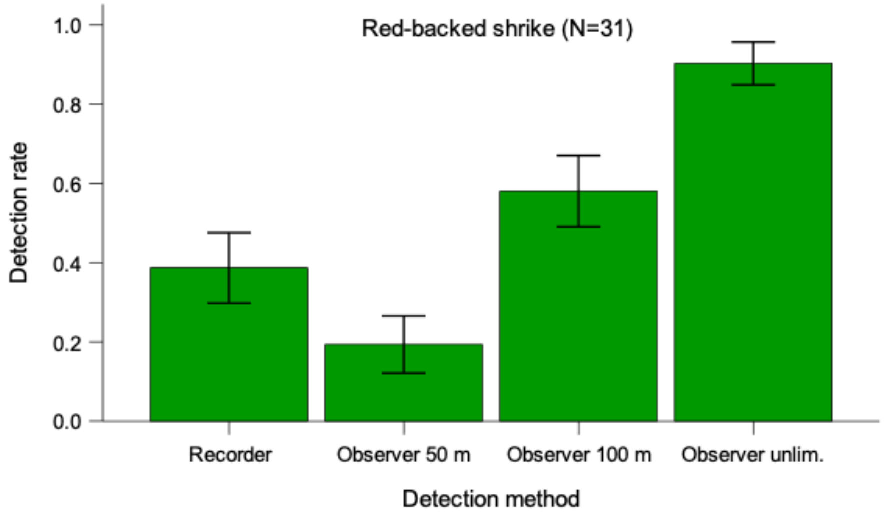

Red-backed shrike

Lanius collurio

	Z	p
R-Ob 50 m	-1.732	0.083
R-Ob 100 m	-1.604	0.109
R-Ob unlm	-3.411	0.001*

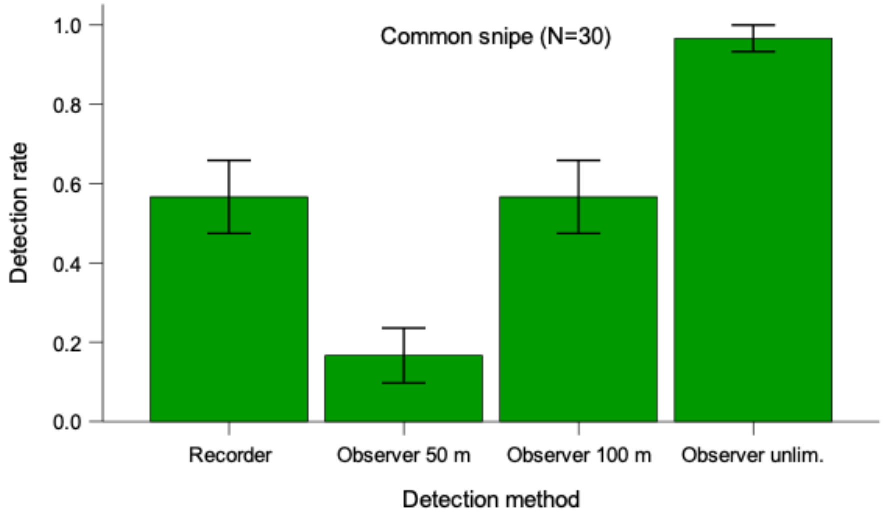

Common snipe

Gallinago gallinago

	Z	p
R-Ob 50 m	-2.828	0.005*
R-Ob 100 m	0.001	1.000
R-Ob unlm	-3.207	<0.001*

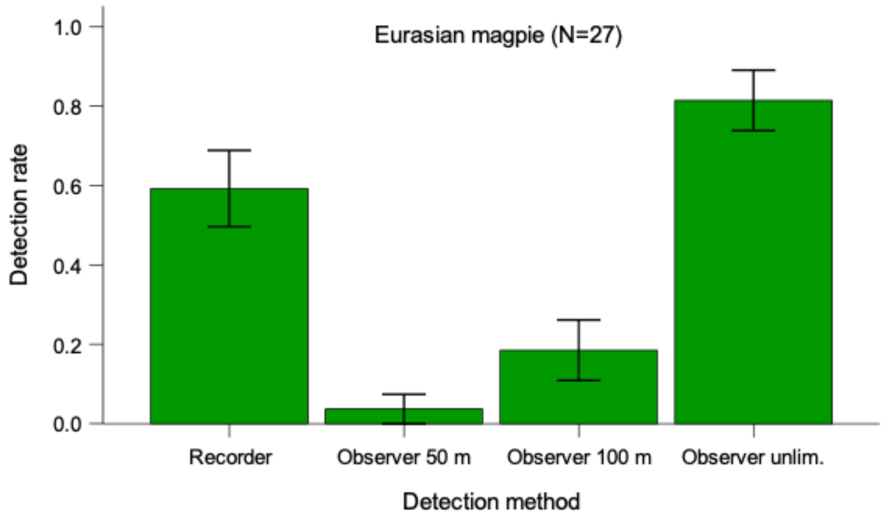

Eurasian magpie

Pica pica

	Z	p
R-Ob 50 m	-3.873	<0.001*
R-Ob 100 m	-2.840	0.005*
R-Ob unlm	-1.500	0.134

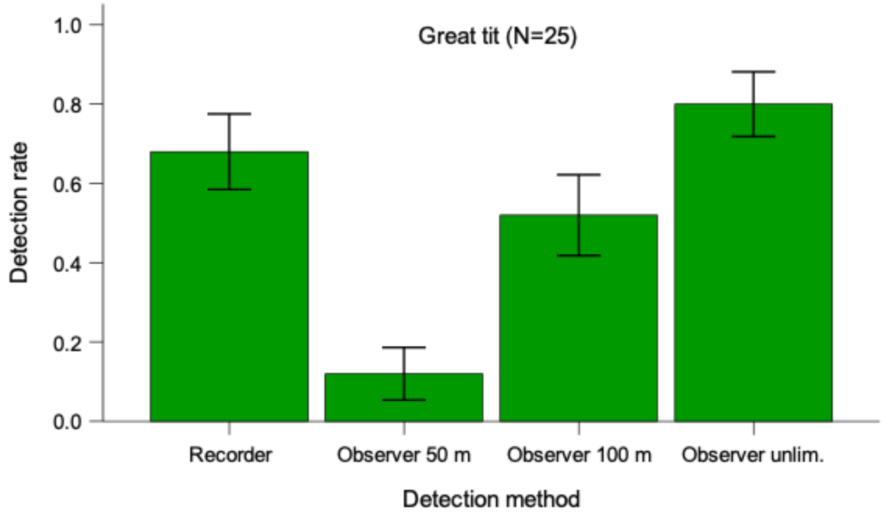

Great tit

Parus major

	Z	p
R-Ob 50 m	-3.300	0.001*
R-Ob 100 m	-1.000	0.317
R-Ob unlm	0.001	0.405

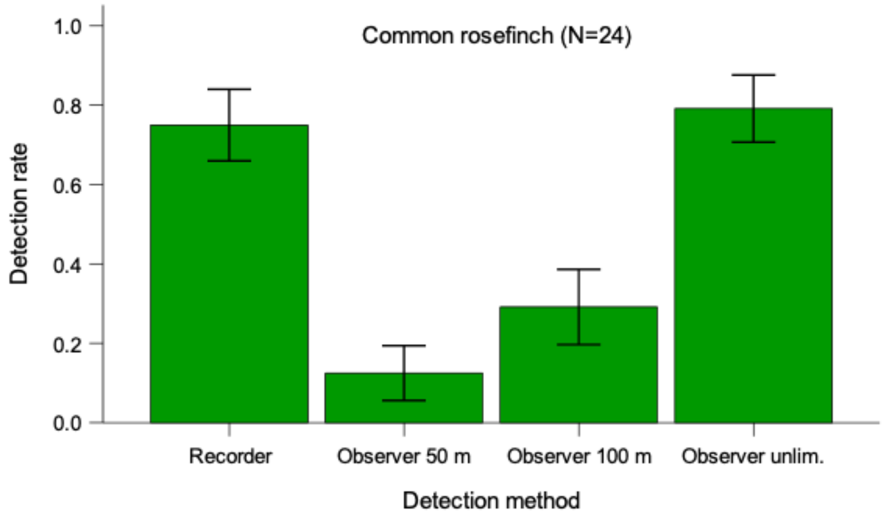

Common rosefinch

Carpodacus erythrinus

	Z	p
R-Ob 50 m	-3.638	<0.000*
R-Ob 100 m	-3.051	0.002*
R-Ob unlm	-0.302	0.763

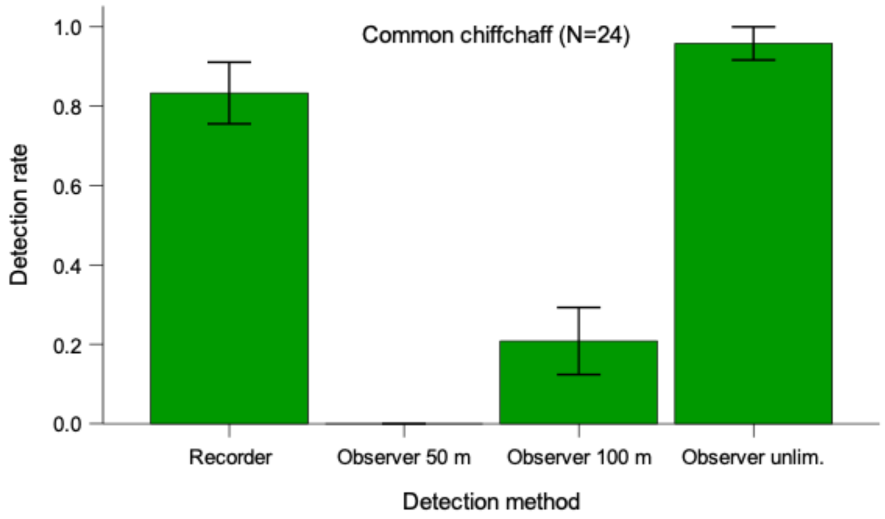

Common chiffchaff

Phylloscopus collybita

	Z	p
R-Ob 50 m	-4.472	<0.001*
R-Ob 100 m	-3.873	<0.001*
R-Ob unlm	-1.342	0.180

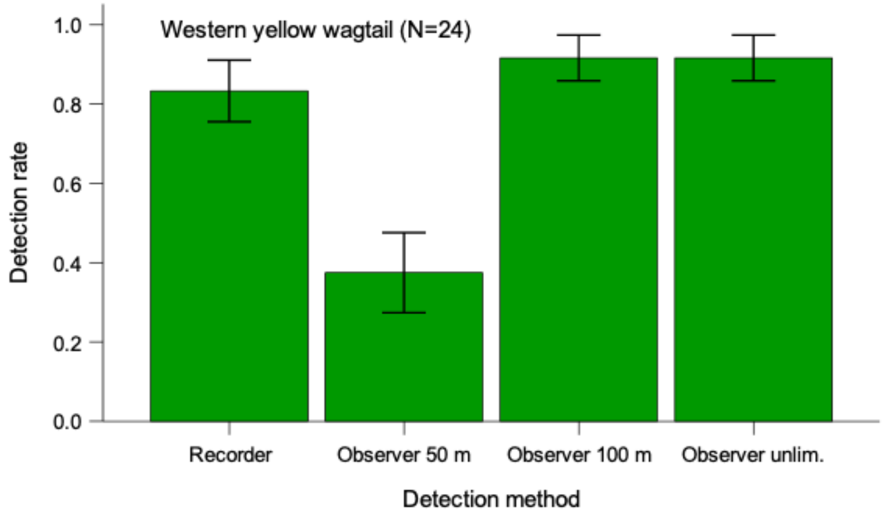

Western yellow wagtail

Motacilla flava

	Z	p
R-Ob 50 m	-3.317	<0.001*
R-Ob 100 m	-0.816	0.414
R-Ob unlm	-.8160	0.414

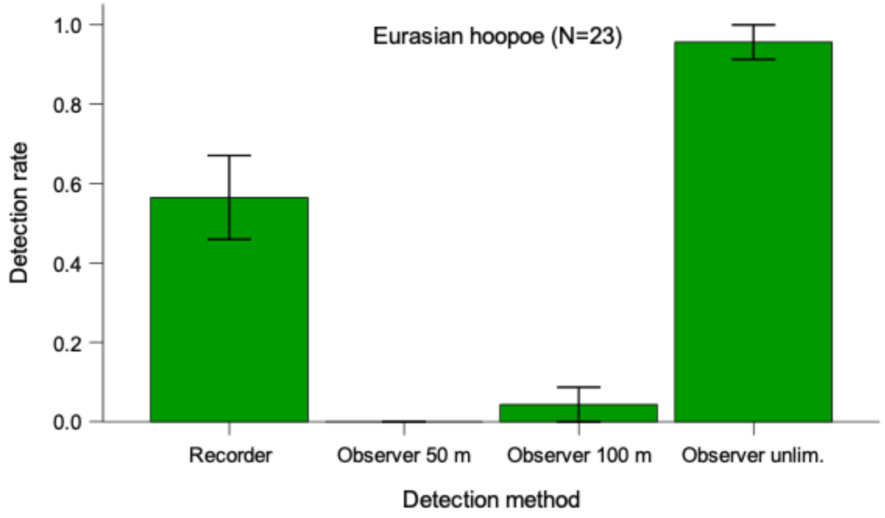

Eurasian hoopoe

Upupa epops

	Z	p
R-Ob 50 m	-3.606	<0.000*
R-Ob 100 m	-3.207	<0.001*
R-Ob unlm	-2.714	0.007*

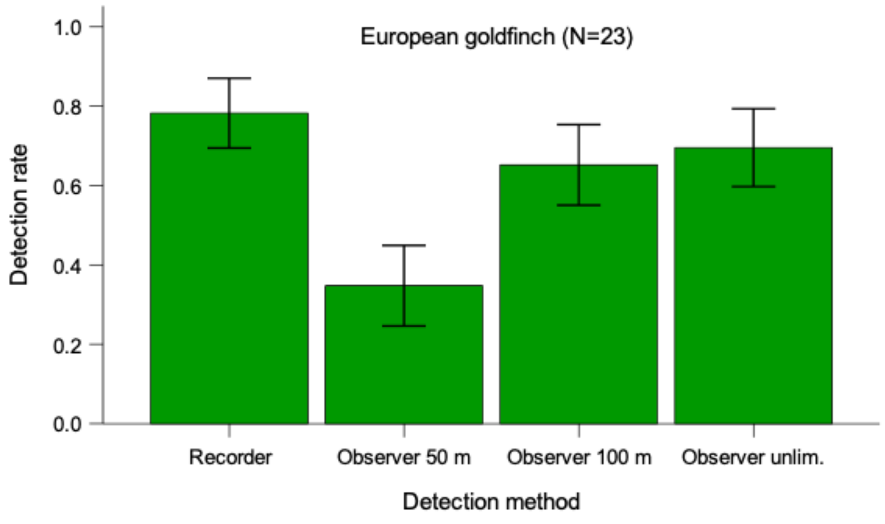

European goldfinch

Carduelis carduelis

	Z	p
R-Ob 50 m	-2.673	0.008*
R-Ob 100 m	-0.832	0.405
R-Ob unlm	-0.577	0.564

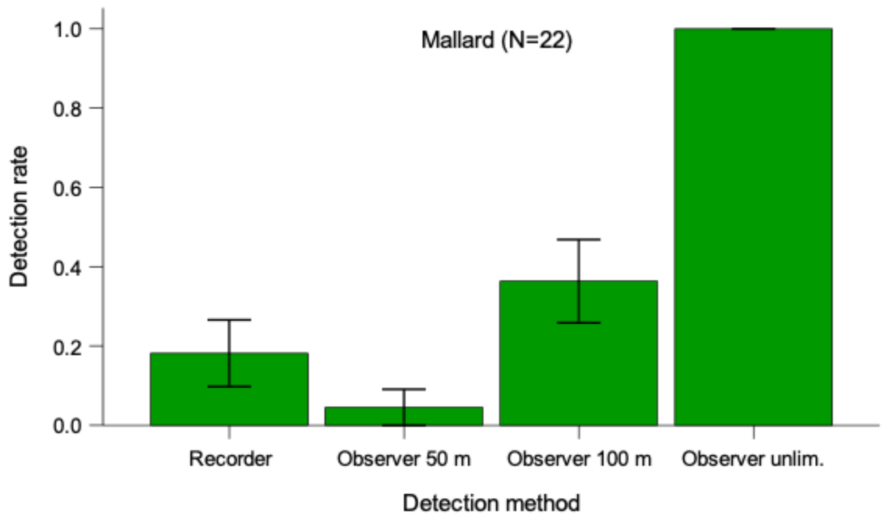

Mallard

Anas platyrhynchos

	Z	p
R-Ob 50 m	-1.342	0.180
R-Ob 100 m	-1.414	0.157
R-Ob unlm	-4.243	<0.001*

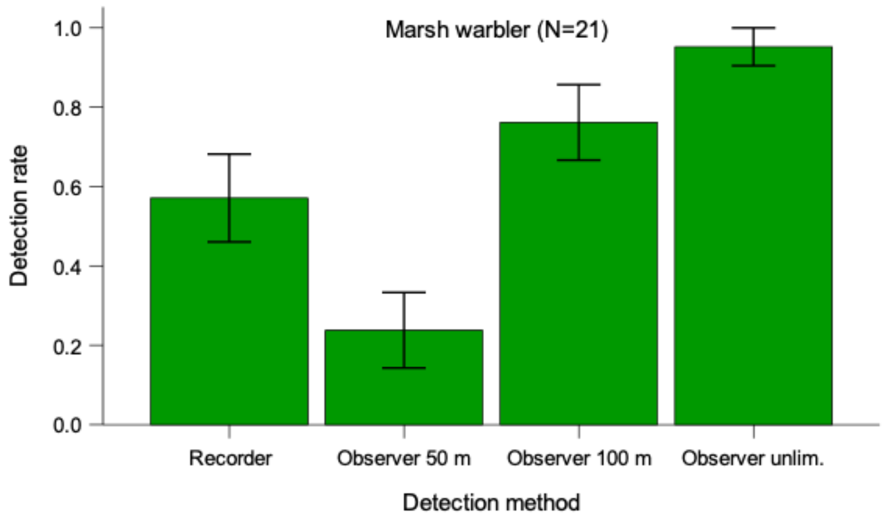

Marsh warbler

Acrocephalus palustris

	Z	p
R-Ob 50 m	-2.333	0.020
R-Ob 100 m	-1.414	0.157
R-Ob unlm	-2.530	0.011*

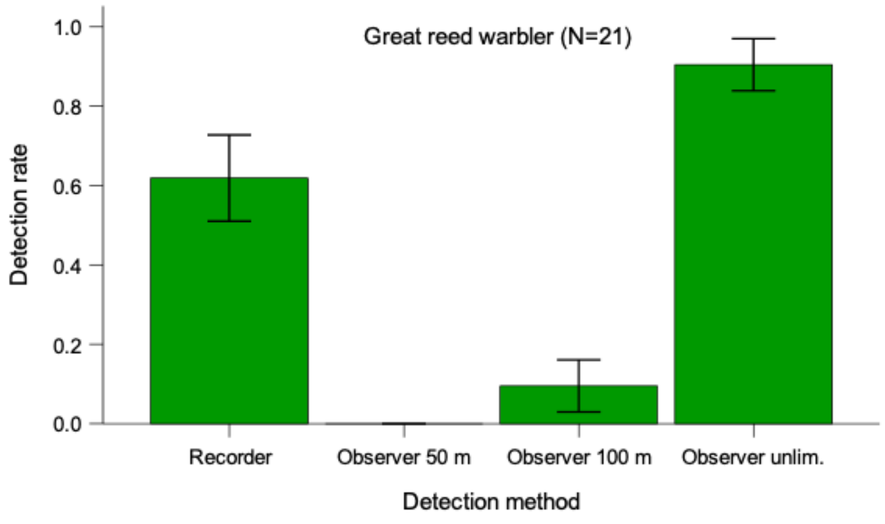

Great reed warbler

Acrocephalus arundinaceus

	Z	p
R-Ob 50 m	-3.606	<0.001*
R-Ob 100 m	-3.051	0.002*
R-Ob unlm	-1.897	0.058

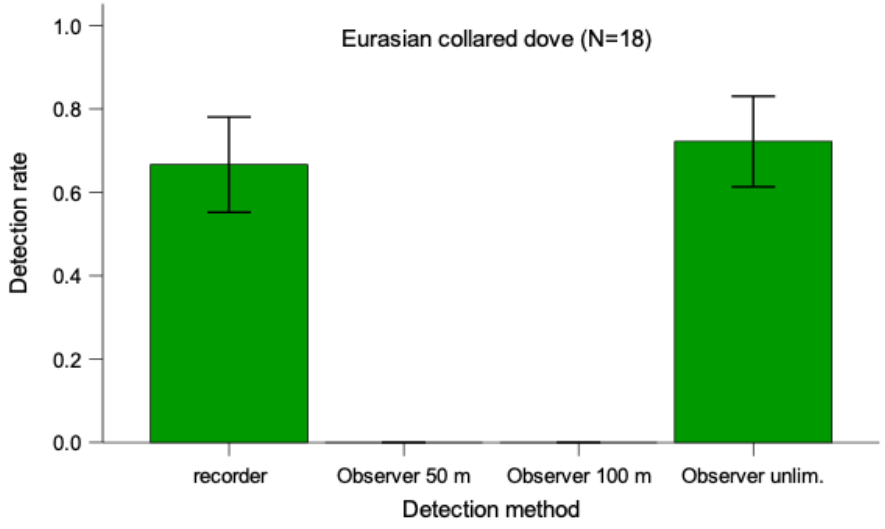

Eurasian collared dove

Streptopelia decaocto

	Z	p
R-Ob 50 m	-3.464	<0.001*
R-Ob 100 m	-3.464	<0.001*
R-Ob unlm	-0.302	0.763

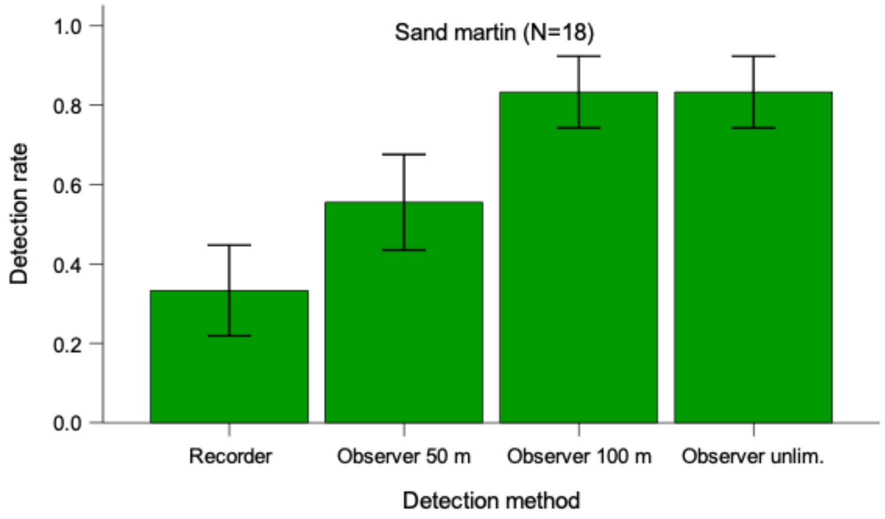

Sand martin

Riparia riparia

	Z	p
R-Ob 50 m	-1.265	0.206
R-Ob 100 m	-2.324	0.020
R-Ob unlm	-2.324	0.020

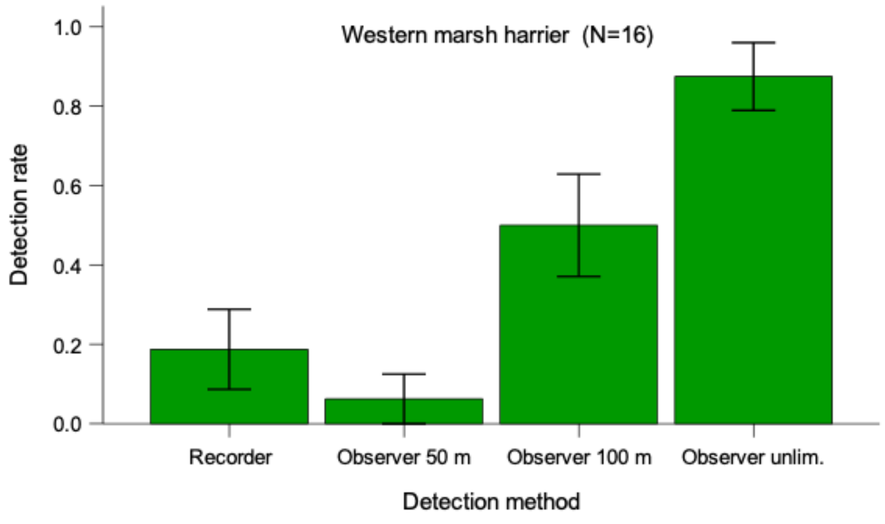

Western marsh harrier

Circus aeruginosus

	Z	p
R-Ob 50 m	-1.414	0.157
R-Ob 100 m	-1.667	0.096
R-Ob unlm	-2.840	0.005*

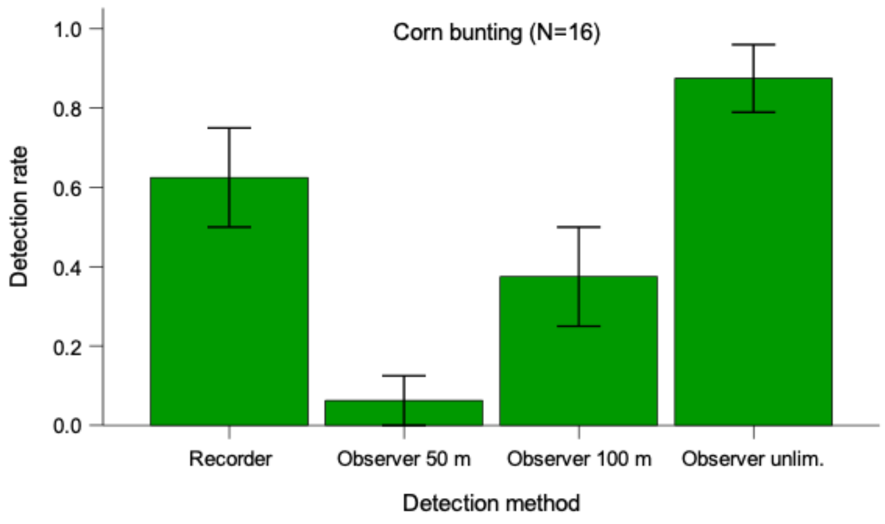

Corn bunting

Emberiza calandra

	Z	p
R-Ob 50 m	-2.714	0.007*
R-Ob 100 m	-1.414	0.157
R-Ob unlm	-1.414	0.157

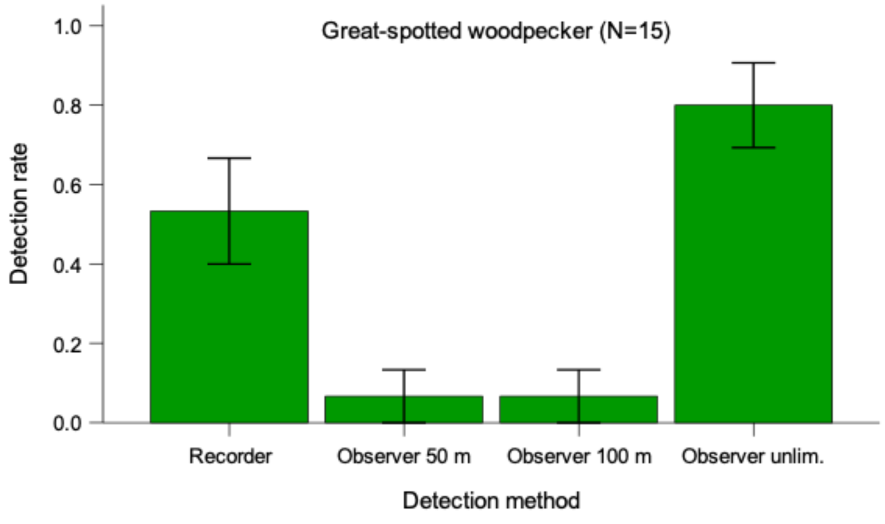

Great-spotted woodpecker

Dendrocopos major

	Z	p
R-Ob 50 m	-2.333	0.020
R-Ob 100 m	-2.333	0.020
R-Ob unlm	-1.265	0.206

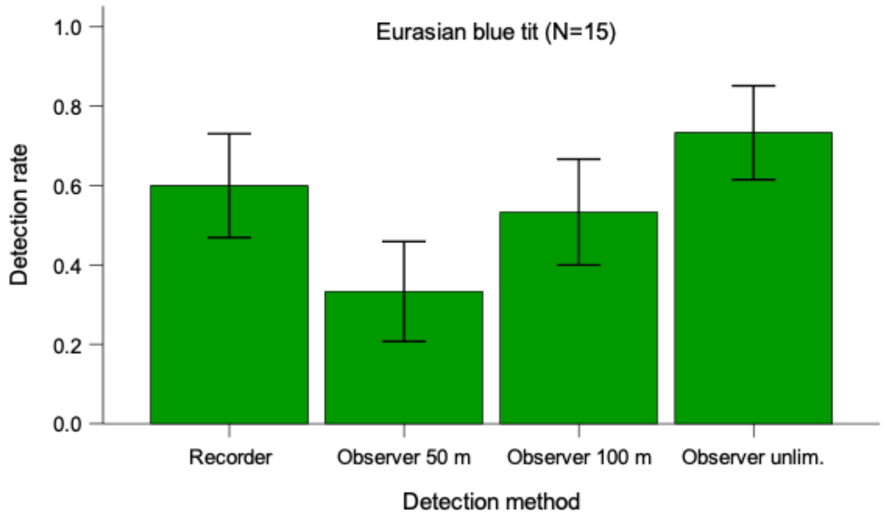

Blue tit

Cyanistes caeruleus

	Z	p
R-Ob 50 m	-1.265	0.206
R-Ob 100 m	-0.302	0.763
R-Ob unlm	-0.632	0.527
