# Supplementary material for: Acoustic approach as an alternative to human-based survey in bird biodiversity monitoring in agricultural meadows
Source: PLoS One. 2022 Apr 8;17(4):e0266557. doi: 10.1371/journal.pone.0266557 (PMC8992991; doi:10.1371/journal.pone.0266557)
Supplement: S2 Table — (PDF) [file pone.0266557.s003.pdf]

S2 Table. List of meadow bird species and farmland bird species.

We applied the list of meadow bird species in Poland according to Tryjanowski et al. 2009. As a farmland birds we defined 22 species that are used to calculate the farmland bird index for Poland. In the study we recorded 17 farmland species and 15 meadow species (bold).

No.	Meadow species	Farmalnd species
1	**Ruff**	**White stork**
2	Dunlin	**Common whitethroat**
3	**Montagu's harrier**	**Northern lapwing**
4	Hen harrier	**Eurasian hoopoe**
5	Black grouse	**Barn swallow**
6	Garganey	Crested lark
7	**Northern lapwing**	**Red-backed shrike**
8	**Corncrake**	European stonechat
9	Great snipe	**European serin**
10	Spotted crake	**Common linnet**
11	**Redshank**	House sparrow
12	**Mallard**	Ortolan bunting
13	**Common snipe**	**Yellow wagtail**
14	**Eurasian curlew**	**Whinchat**
15	**Marsh warbler**	**Corn bunting**
16	**Yellow wagtail**	**Common kestrel**
17	Northern shoveler	**Black-tailed godwit**
18	**Whinchat**	**Skylark**
19	Pintail	**Common starling**
20	**Black-tailed godwit**	**Meadow pipit**
21	**Skylark**	**Yellowhammer**
22	**Meadow pipit**	European turtle dove
23	**Grasshopper warbler**	
24	Short-eared owl	
25	Aquatic warbler	

Tryjanowski P, Kuźniak S, Kujawa K, Jerzak L. 2009. Ekologia ptaków krajobrazu rolniczego (Ecology of farmland birds). Bogucki wydawnictwo Naukowe, Poznań, Polska. [in Polish]
