# Supplementary material for: Acoustic approach as an alternative to human-based survey in bird biodiversity monitoring in agricultural meadows
Source: PLoS One. 2022 Apr 8;17(4):e0266557. doi: 10.1371/journal.pone.0266557 (PMC8992991; doi:10.1371/journal.pone.0266557)
Supplement: S4 Table — (PDF) [file pone.0266557.s005.pdf]

S4 Table. List of bird species detected only by recorder and only by human-observer. Table shows: the number of surveys in which species was observed, the number of recording sites in which species was observed, the number of detections by a recorder, the number of detections by a human-observer.

List of bird species detected only by recorders				
Species (English name)	Species (Latin name)	Number of surveys	Number of recording sites	Number of detections by recorder
Willow tit	Poecile montanus	1	1	1
Lesser spotted woodpecker	Dryobates minor	1	1	1
Eurasian nuthatch	Sitta europaea	1	1	1
Marsh tit	Poecile palustris	1	1	1
Common greenshank	Tringa nebularia	3	3	3
List of bird species detected only by human-observers				
Species (English name)	Species (Latin name)	Number of surveys	Number of recording sites	Number of detections by human-observer
White-tailed eagle	Haliaeetus albicilla	1	1	1
Black kite	Milvus migrans	1	1	1
Great cormorant	Phalacrocorax carbo	1	1	1
Red crossbill	Loxia curvirostra	1	1	1
European serin	Serinus serinus	1	1	1
Grey partridge	Perdix perdix	1	1	1
Peregrine falcon	Falco peregrinus	1	1	1
European honey buzzard	Pernis apivorus	1	1	1
River warbler	Locustella fluviatilis	2	2	2
Eurasian collared dove	Streptopelia decaocto	2	2	2
Montagu's harrier	Circus pygargus	3	3	3
Great egret	Ardea alba	3	3	3
Lesser spotted eagle	Clanga pomarina	3	3	3
Great bittern	Botaurus stellaris	4	4	4
Common tern	Sterna hirundo	4	4	4
Green sandpiper	Tringa ochropus	4	4	4
Stock dove	Columba oenas	4	4	4
Ruff	Calidris pugnax	6	6	6
Common kestrel	Falco tinnunculus	7	7	7
Grey heron	Ardea cinerea	13	13	13
